# Chemical, computational and functional insights into the chemical stability of the Hedgehog pathway inhibitor GANT61

**DOI:** 10.1080/14756366.2017.1419221

**Published:** 2018-01-17

**Authors:** Andrea Calcaterra, Valentina Iovine, Bruno Botta, Deborah Quaglio, Ilaria D’Acquarica, Alessia Ciogli, Antonia Iazzetti, Romina Alfonsi, Ludovica Lospinoso Severini, Paola Infante, Lucia Di Marcotullio, Mattia Mori, Francesca Ghirga

**Affiliations:** a Department of Chemistry and Technology of Drugs, Sapienza University of Rome, Rome, Italy;; b Department of Molecular Medicine, Sapienza University of Rome, Rome, Italy;; c Center for Life Nano Science@Sapienza, Istituto Italiano di Tecnologia, Rome, Italy;; d Pasteur Institute/Cenci Bolognetti Foundation, Sapienza University of Rome, Rome, Italy

**Keywords:** GANT61, Hedgehog pathway, Gli inhibitor, chemical stability, bioactive form

## Abstract

This work aims at elucidating the mechanism and kinetics of hydrolysis of GANT61, the first and most-widely used inhibitor of the Hedgehog (Hh) signalling pathway that targets Glioma-associated oncogene homologue (Gli) proteins, and at confirming the chemical nature of its bioactive form. GANT61 is poorly stable under physiological conditions and rapidly hydrolyses into an aldehyde species (GANT61-A), which is devoid of the biological activity against Hh signalling, and a diamine derivative (GANT61-D), which has shown inhibition of Gli-mediated transcription. Here, we combined chemical synthesis, NMR spectroscopy, analytical studies, molecular modelling and functional cell assays to characterise the GANT61 hydrolysis pathway. Our results show that GANT61-D is the bioactive form of GANT61 in NIH3T3 Shh-Light II cells and SuFu^−/−^ mouse embryonic fibroblasts, and clarify the structural requirements for GANT61-D binding to Gli1. This study paves the way to the design of GANT61 derivatives with improved potency and chemical stability.

## Graphical abstract

**Figure:**
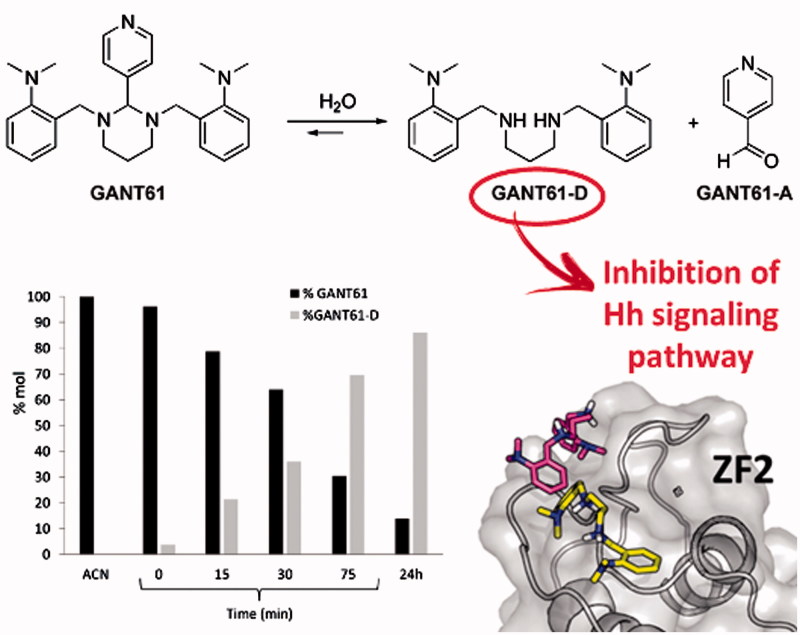

## Introduction

One of the most challenging tasks in drug discovery is the identification of the active species responsible for the observed biological activity of a drug or a drug candidate (thereafter referred as the bioactive form). In fact, any molecular entity may undergo structural modifications due to the exposure to different cellular environments, metabolic enzymes, reactions with redox species, and so on. In this regard, understanding the metabolic or chemical stability of a bioactive compound may help to unravel its mechanism of action and to optimise pharmacokinetics properties, which increases the success rate of a drug discovery campaign^1^ ^,^ ^2^.

In the last 10 years, many efforts have been spent by our own and other research groups on the identification of small molecule inhibitors of the Hedgehog (Hh) signalling pathway^3–8^, which plays a pivotal role in the initiation, proliferation, invasion and metastasis of a wide variety of cancers including basal cell carcinoma (BCC), medulloblastoma^9–11^, rhabdomyosarcoma^12^ ^,^ ^13^, pancreatic^14^, colorectal^15^, metastatic prostate^16^, small-cell lung^17^, breast^18^ carcinomas, and malignant gliomas. The Hh pathway is also implicated in the regulation and maintenance of cancer stem cells (CSCs), providing a link between the Hh signalling in the regulation of normal stem cells and its role in CSCs maintenance^19–22^. Noteworthy, targeting CSCs has recently emerged as a profitable anticancer strategy endowed with impressive therapeutic implications and challenges^23^ ^,^ ^24^.

Smoothened receptor (Smo) is the most widely appreciated drug target of the Hh signalling pathway. Two small molecule antagonists of Smo have been approved by the FDA (namely, Vismodegib in 2012 and Sonidegib in 2015) for the treatment of metastatic or locally advanced BCC^25^ ^,^ ^26^, while a number of clinical trials are currently running on additional chemotypes of Smo antagonists^27^ ^,^ ^28^. However, drug-resistant mutations of Smo sequence^29–31^, as well as Smo-independent Hh pathway activation have been highlighted during the treatment with Vismodegib or other clinical candidates^32^ ^,^ ^33^, thus raising the need to identify novel leads capable to act on the drug-resistant forms of Smo and/or to block the pathway downstream or independently by Smo^34^. In this regard, the Glioma-associated oncogene homologue 1 (Gli1) is the final effector of the Hh pathway and has emerged as an alternative and more promising target than Smo^6^.

**Figure 1.**
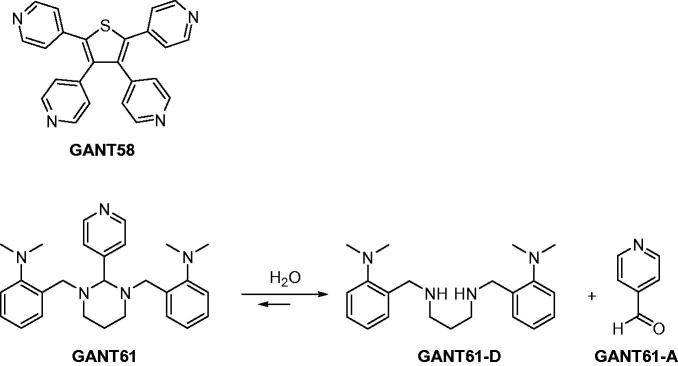
Chemical structures of two Gli antagonists GANT58 and GANT61 discovered in 2007 by Lauth et al. and GANT61 hydrolysis products GANT61-D and GANT61-A.

By integrating multidisciplinary efforts in a concerted strategy, recently we proved that Gli1/DNA interaction is a druggable target for the treatment of Hh-dependent tumours^8^. We also identified the naturally occurring isoflavone Glabrescione B (GlaB) as the first small molecule capable of impairing Gli1 activity by directly interfering with its binding to DNA^8^. However, for the sake of clarity GANT61 (Figure 1) has been the first small molecule reported to inhibit Gli1 activity in living cells. It was discovered in 2007 by Lauth et al. in a cell-based screen for small molecule inhibitors of Gli-mediated transcription, together with GANT58 (Figure 1)^35^. These two molecules proved to inhibit both Gli1- and Gli2-mediated gene transactivation in a dose-dependent manner, with an IC_50_ value of about 5 µM in cellular assays.

A subsequent study has highlighted the possible chemical instability of GANT61 by designing a GANT61 diamine derivative (GANT61-D, Figure 1) that has shown Hh inhibitory efficacy comparable to the parent prodrug GANT61^36^. The same authors have also underlined the chemical instability of GANT61 in aqueous buffers^37^, suggesting that GANT61-D and the pyridine 4-carboxyaldehyde (GANT61-A) are formed according to the pathway shown in Figure 1.

GANT61-D has been the subject of another recent study aimed at investigating its mode and specificity of binding to Gli proteins and to further corroborate the druggability of Gli in cancer^38^. Molecular docking simulations have identified the putative GANT61-D binding site within residues E119 and E167, although the authors have carried out molecular docking on the neutral form of the GANT61-D that is expected to be less abundant than the corresponding mono- and di-protonated forms at physiological pH values.

Even though these prior works^36–38^ synergistically substantiated that GANT61-D is the bioactive form of GANT61, they fail to provide clear and detailed information on the mechanism and kinetics of GANT61 hydrolysis, thus limiting the further development of GANT61 as anticancer lead. To fill this gap, and to pursue our scientific interest in developing Hh-targeting small molecules, here we combined multiple approaches including chemical synthesis, computational modelling, analytical tools and functional studies to provide additional insights into the chemical stability and Hh inhibitory activity of GANT61. To this end, we synthesised both GANT61 and GANT61-D in order to provide sufficient amounts of pure compounds for analytical and functional assays. Afterwards, GANT61 chemical stability was monitored by NMR spectroscopy and hydrophilic interaction chromatography (HILIC), whereas molecular modelling was used to evaluate the mode of binding of GANT61-D in its neutral as well as multiple ionisation states to Gli1 zinc finger domain (Gli1ZF). Hh pathway inhibition by GANT61 and GANT61-D was finally evaluated in NIH3T3 Shh-Light II cells stably incorporating a Gli-responsive firefly luciferase reporter (Gli-RE), and in SuFu^−/−^ mouse embryonic fibroblasts (MEFs) to monitor effects on the Hh signalling at a downstream level.

## Methods

### General experimental procedures

Commercially available reagents were supplied by Sigma-Aldrich (St. Louis, MO) and used without further purification. A sample of GANT61 was purchased from Tocris Bioscience (Bristol, UK) for comparison with the newly synthesised sample. Dry solvents were purchased from Sigma-Aldrich except for ethanol, which was dried by distillation from I_2_/Mg.

^1^H and ^13^C NMR spectra have been acquired with a Bruker Avance 400 spectrometer operating at 400.13 and 100.6 MHz, respectively, at 300 K in CDCl_3_, MeOD or DMSO-d_6_, using 5-mm diameter glass tubes.

^1^H NMR spectra of GANT61 for kinetic studies were recorded at 310 K in EtOH-d_6_/PBS-*d*(D_2_O) at 50:50 ratio (v/v). The deuterated PBS buffer was prepared by mixing 0.066 M stock solution of K_2_DPO_4_ (98 atom % D) and KD_2_PO_4_ (98 atom % D) prepared in D_2_O to the correct pD = 7.4, diluting the solution to the proper phosphate buffer concentration reported for PBS, and then adding NaCl and KCl to the final solution in order to reach the concentration of 0.137 M and 0.0027 M, respectively. The mixture was thoroughly equilibrated for ∼1 h before being used for NMR measurements. The resulting pD was 7.4 (assuming that pD = pH meter reading +0.4)^39^, otherwise it was adjusted to 7.4 by addition of 37% w/w DCl or 40% w/w NaOD solutions in D_2_O.

Chemical shifts were expressed in ppm and coupling constants (*J*) in Hertz (Hz), approximated to 0.1 Hz. Residual solvent peak was used as an internal reference for ^1^H NMR and ^13^C NMR spectra. Data for ^1^H NMR are reported as follows: chemical shift, multiplicity (ovrlp = overlapped, s = singlet, d = doublet, t = triplet, p = pentuplet, m = multiplet, dd = doublet of doublets, dt = doublet of triplets, td = triplet of doublets, ABq = AB quartet), coupling constant, integral. Spectra were processed with the program MestReNova version 6.0.2–5475, FT and zero filling at 64 K.

Mass spectra were obtained using a Thermo Finnigan LCQ Deca XP Plus mass spectrometer equipped with an electrospray ionisation (ESI) source and a Fleet ion-trapanalyser; capillary temperature 275 °C, spray voltage 5.0 kV (positive mode), sheath gas (N_2_) 25 arbitrary units, capillary voltage 40 V, tube lens 15 V.

Analytical liquid chromatography was performed using a Waters-1525 HPLC system equipped with an UV detector (Waters 2487) and an evaporative light scattering detector (ELSD) (SEDEX) detector. The column used was the Acclaim HILIC-10, 3 µm (150 × 4.6 mm I.D.), purchased from Thermo Scientific (Waltham, MA). Eluent: CH_3_CN/NH_4_OAc 100 mM (pH = 4.5) at 95:5 ratio (v/v). Flow-rate: 1.0 ml/min, room temperature. Detection: UV at 254 nm, and ELSD (*P* = 3.0 bar, T = 60 °C). The crude product purifications were carried out on silica column chromatography using Silica Gel Fluka 60 Å (0.063–0.200 mm, 70–230 mesh). GANT61 was purified by column chromatography on aluminium oxide active basic EMD Millipore (0.063–0.200 mm, 70–230 mesh, pH = 9–10.5).

### Synthesis of 2-(dimethylamino)benzaldehyde (2)

Commercially available 2-fluorobenzaldehyde (1) (0.45 g, 0.38 ml, 3.62 mmol) and K_2_CO_3_ (1.0 g, 7.24 mmol) were introduced in a sealed tube under an argon atmosphere. Anhydrous DMSO (0.38 ml) and a 2 M solution of (CH_3_)_2_NH in THF (2.0 ml, 4.0 mmol) were added and the mixture was heated to reflux temperature (about 100 °C) for 3 h. Then, after cooling, another aliquot (2.0 ml, 4.0 mmol) of 2 M solution of (CH_3_)_2_NH in THF was added and the mixture was refluxed for other 3 h. The last addition of the (CH_3_)_2_NH solution (2.0 ml, 4.0 mmol) was made before refluxing the mixture overnight. The reaction mixture was poured into water and extracted with CH_2_Cl_2_. Organic layers were collected, dried over Na_2_SO_4_ and concentrated *in vacuo* to give a pale yellow oil. The crude product was purified by column chromatography using silica gel and 5% ethyl acetate/*n*-hexane as eluent to obtain 2-(dimethylamino)benzaldehyde (2) in 65% yield (0.35 g, 2.35 mmol). Pale yellow oil; ^1^H NMR (400.13 MHz, CDCl_3_) δ 10.20 (s, 1H), 7.74 (dd, *J*_1_ = 7.6 Hz, *J*_2_ = 1.6 Hz, 1H), 7.46–7.41 (m, 1H), 7.02 (d, *J* = 8.0 Hz, 1H), 6.97 (t, *J* = 7.6 Hz, 1H), 2.89 (s, 6H); ^13^C NMR (100.6 MHz, CDCl_3_) δ 191.2, 155.9, 134.6, 131.1, 127.1, 120.8, 117.7, 45.5; ESI-MS (pos.) *m/z* = 150 ([M + H]^+^).

### Synthesis of N^1^,N^3^-(bis-2-isopropylbenzyl)propane-1,3-diamine (GANT61-D)

In a three-necked round bottom flask, equipped with a Dean–Stark separator fulfilled with molecular sieves (pore size 3 Å) and anhydrous benzene, were introduced benzaldehyde **2** (0.166 g, 1.11 mmol), anhydrous benzene (15 ml) and 1,3-diaminopropane (0.041 g, 46 µl, 0.556 mmol), freshly distilled from molecular sieves. The mixture was heated at reflux temperature and stirred overnight. The reaction mixture, monitored by TLC (eluent: *n*-hexane/ethyl acetate = 90:10), showed the partial disappearance of the starting material after 12 h. After that time, the solvent was removed under reduced pressure and the residue was dissolved in 10 ml of dry ethanol. After cooling the solution to 0 °C, NaBH_4_ (0.083 g, 2.226 mmol) was added and the mixture was stirred for 30 min. Then, the reaction was quenched with a saturated aqueous NH_4_Cl solution and most of the solvent (ethanol) was removed under reduced pressure. The aqueous residue was washed with ethyl acetate (3 × 15 ml) and the organic layers were discarded. 2 M NaOH was added to the aqueous solution until pH 14 and a white precipitate rapidly appeared. The mixture was thus extracted with ethyl acetate (4 × 15 ml) and the collected organic layers were dried over Na_2_SO_4_ and concentrated *in vacuo* to give a yellow oil. The crude product was purified by column chromatography using silica gel and the mixture MeOH/Et_3_N/CH_2_Cl_2_ 10:5:85 as eluent to obtain the pure GANT61-D in 60% yield (0.11 g, 0.34 mmol). Oily transparent or yellowish liquid; ^1^H NMR (400.13 MHz, MeOD) δ 7.28–7.17 (m, 6H), 7.04 (td, *J*_1_ = 7.2 Hz, *J*_2_ = 1.2 Hz, 2H), 3.86 (s, 4H), 2.65 (ovrlp s, 12H), 2.68–2.60 (ovrlp m, 4H), 1.75 (p, *J* = 7.1 Hz, 2H). ^13^C NMR (100.6 MHz, MeOD) δ 154.3, 134.4, 131.0, 129.4, 125.0, 121.0, 51.0, 48.2, 45.4, 29.5. ESI-MS (pos.) *m/z* = 341 ([M + H]^+^), 171 ([M + 2H]^2+^).

### Synthesis of GANT61

In a three-necked round bottom flask were introduced GANT61-D (0.051 g, 0.15 mmol) in anhydrous THF (0.5 ml) and commercially available pyridine 4-carboxyaldehyde (GANT61-A; 0.016 g, 0.15 mmol) in anhydrous THF (1.0 ml). The reaction mixture was heated at reflux temperature and stirred for 18 h. The solvent was removed under reduced pressure and the crude residue was purified by column chromatography using basic alumina and ethyl acetate/*n*-hexane as eluent to obtain GANT61 in 90% yield (0.058 g, 0.135 mmol). White solid; ^1^H NMR (400.13 MHz, DMSO-d_6_) δ 8.55 (dd, *J* = 4.5, 1.3 Hz, 2H), 7.67 (dd, *J* = 4.5, 1.3 Hz, 2H), 7.47 (dd, *J* = 4.5, 1.3 Hz, 2H), 7.15 (m, 2H), 7.06–6.98 (m, 4H), 4.01 (s, 1H), 3.40, 3.51 (ABq, *J*_AB_ = 14.2 Hz, 4H), 2.81 (td, *J* = 11.5, 3.7 Hz, 2H), 2.50 (ovrlp s, 12H), 2.18 (p, *J* = 6.3 Hz, 2H), 1.62–1.54 (m, 2H). ^13^C NMR (100.6 MHz, DMSO-d_6_) δ 152.5, 150.6, 149.5, 132.9, 128.8, 127.1, 124.4, 122.8, 118.8, 85.3, 52.00, 49.9, 44.7, 22.51. ESI-MS (pos.) *m/z* = 431 ([M + H]^+^), 215.7 ([M + 2H]^2+^).

### Molecular modelling

The crystallographic structure of Gli1ZF in complex with DNA (PDB ID: 2GLI)^40^ was used as a rigid receptor in molecular docking simulations performed with AutoDock4.2^41^. Before doing docking, DNA atoms and water molecules were manually removed, whereas cobalt ions present in the X-ray structure were substituted with Zn(II) ions as in the physiologically relevant human Gli1. For grid generation, a box of 80 × 70 × 80 points with a grid spacing of 0.5 Å that covered the whole Gli1ZF accessible surface was centred on the mass centre of the protein. An arbitrary +1 charge was set to Zn(II) ions within each ZF, taking into consideration that coordination by amino acids decreases the total point charge of a metal ion as observed in prior studies^42–45^. Docking calculations were performed with AutoDock using the Lamarckian Genetic Algorithm by running 100 GA_RUNS for each molecule and keeping all other parameters at their default value. Electrostatic properties of Gli1ZF were evaluated by solving the Poisson–Boltzmann equation using APBS^46^.

Docking complexes were further relaxed by energy minimisation in explicit water solvent (TIP3P type) with Amber12^47^. The ff12 and the GAFF force fields were used for simulating proteins and ligands, respectively. As in docking simulations, an arbitrary charge of +1 and was assigned to Zn(II) ions, which were treated by a non-bonded approach (harmonic restraint of 10 kcal/(mol•Å^2^)). Energy minimisation was performed as follows, in agreement with prior works^42^ ^,^ ^48–51^: (i) water molecules were energy minimised for 100 steps with a steepest-descent algorithm (SD) and subsequent 300 steps with a conjugate gradient algorithm (CG) while keeping the solute as frozen; (ii) the solvated solute was energy minimised for 1000 steps SD and 9000 steps CG. After energy minimisation, ligand theoretical affinity was calculated by means of the Molecular Mechanics Generalised Born Surface Area (MM-GBSA) method^52^ ^,^ ^53^ and the XSCORE function^54^. Prediction of pKa of GANT61-D was performed by MoKa (Molecular Discovery Ltd.)^55^.

### Hh-dependent luciferase reporter assay

The luciferase assay was performed in NIH3T3 Shh-Light II cells, stably incorporating a Gli-responsive luciferase reporter and the pRL-TK Renilla (normalisation control). The Shh-Light II cells were cultured in DMEM plus 10% FBS and then treated for 48 h with SAG alone (200 nM, Alexis Biochemicals, Farmingdale, NY) or in combination with GANT61 or GANT61-D.

Luciferase and *Renilla* activity were assayed with a dual-luciferase assay system according to the manufacturer’s instruction (Biotium Inc., Hayward, CA). Results are expressed as luciferase/*Renilla* ratios and represent the mean ± SD of three independent experiments, each performed in triplicate.

### mRNA expression analysis

Total RNA was isolated with Trizol (Invitrogen/Life Technologies, Carlsbad, CA) from SuFu^−/−^ MEFs cultured in DMEM plus 10% FBS and then treated for 24 h with GANT61 or GANT61-D. RNA was then reverse transcribed with SensiFAST cDNA Synthesis Kit (Bioline Reagents Limited, London, UK). Quantitative real-time PCR (Q-PCR) analysis of Gli1, β-2 microglobulin and HPRT mRNA expression was performed on each cDNA sample using the VIIA7 Real-Time PCR System employing Assay-on-Demand Reagents (Life Technologies, Carlsbad, CA). A reaction mixture containing cDNA template, SensiFAST Probe Lo-ROX Kit (Bioline Reagents Limited) and primer-probe mixture was amplified using FAST Q-PCR thermal cycler parameters. Each amplification reaction was performed in triplicate and the average of the three threshold cycles was used to calculate the amount of transcript in the sample (using SDS version 2.3 software). mRNA quantification was expressed, in arbitrary units, as the ratio of the sample quantity to the quantity of the calibrator. All values were normalised with two endogenous controls, β-2 microglobulin and HPRT, which yielded similar results.

## Results

### Chemical synthesis of GANT61 and GANT61-D

**Scheme 1.**
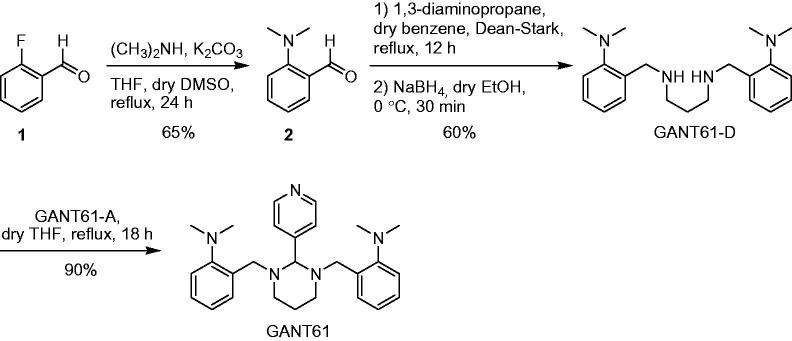
Synthetic pathway to obtain both GANT61-D and GANT61.

As recently disclosed by Chenna et al., GANT61-D (Figure 1) is an intermediate in the synthetic pathway to GANT61^56^. Therefore, here we applied a similar chemical synthesis protocol with slight modifications (see Scheme 1) to obtain both compounds in good yields for analytical and functional studies.

Briefly, nucleophilic aromatic substitution of commercially available 2-fluorobenzaldehyde (**1**) with dimethylamine led to 2-(dimethylamino)benzaldehyde (**2**) in 65% yield. Afterwards, **2** was converted into the intermediate diimine by treatment with 1,3-diaminopropane. Reduction *in situ* with sodium borohydride afforded GANT61-D in 39% overall yield from 2-fluorobenzaldehyde (**1**). To obtain GANT61, GANT61-D was condensed with commercially available GANT61-A (see Scheme 1), thus yielding the target compound in 35% overall yield. The structures of GANT61, GANT61-D and **2** were confirmed by ^1^H and ^13^C NMR spectroscopy and by ESI mass spectrometry (ESI-MS). The NMR spectra of chemically synthesised GANT61 were superimposable with those of a sample of GANT61 purchased from Tocris Bioscience and used for comparison (data not shown).

### Chemical stability of GANT61 checked by NMR spectroscopy

**Figure 2.**
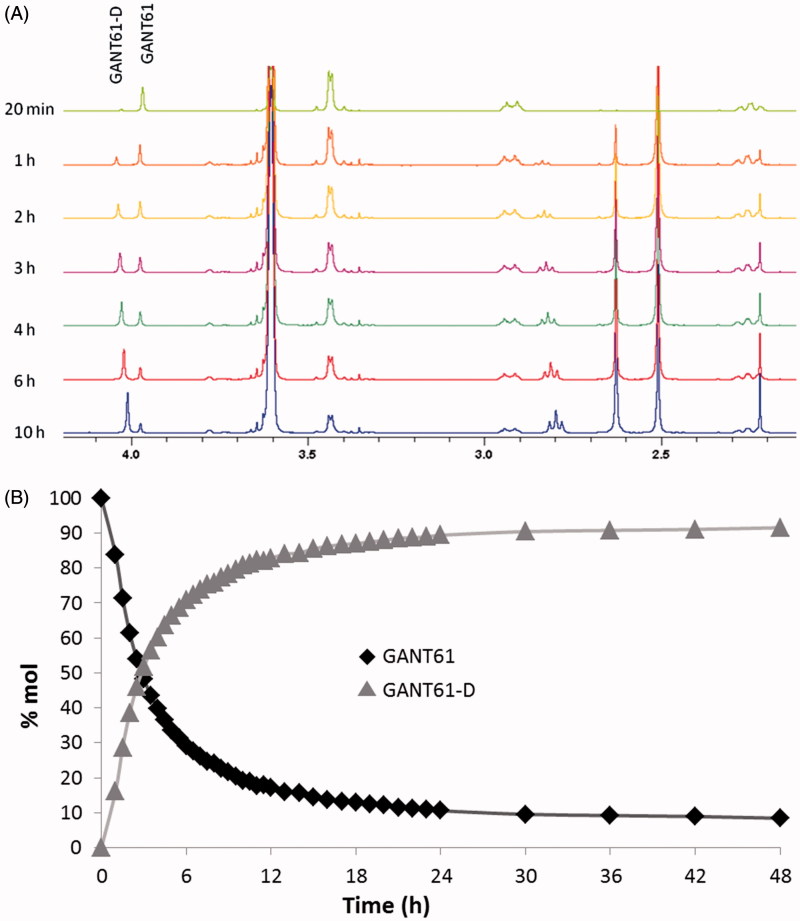
GANT61 hydrolysis in EtOH-d_6_/PBS-*d*(D_2_O) 50:50 v/v at 37 °C monitored by ^1^H NMR spectroscopy. **(A)** Time evolution of the ^1^H NMR spectrum in the aliphatic signals region (2–4.2 ppm); peaks corresponding to the monitored proton of GANT61 and GANT61-D are labelled. **(B)** Evolution of the normalised areas of the selected ^1^H NMR signals for GANT61 and GANT61-D monitored up to 48 h incubation.

NMR is a powerful tool to monitor the chemical stability of bioactive substances in solution and to clarify their possible degradation pathway^57^. Due to limited water solubility, NMR-based kinetic studies of GANT61 were performed in a 1:1 mixture of EtOH-*d*_6_ and deuterated PBS buffer prepared by mixing Na_2_DPO_4_ and KD_2_PO_4_ in D_2_O and adding proper amounts of NaCl and KCl (pD = 7.4). In addition, and with the aim to reproduce as much as possible the physiological pathway of GANT61 hydrolysis in living cells, NMR experiments were performed at 310 K (37 °C). The singlet centred at δ = 3.97 ppm in the ^1^H NMR spectrum of a freshly prepared solution of GANT61 was attributed to the aminalic proton (N-CH-N) and was selected as a probe to monitor the kinetics of hydrolysis of GANT61 (Figure 2(A)). In fact, this proton resonates in a rather clean and empty region of the NMR spectrum and is thought to be included in GANT61-A upon hydrolysis of GANT61 based on the pathway described in Figure 1. The intensity of this signal (normalised with respect to the adjacent increasing signal) proved to decrease during the time (Figure 2(A)), which indicates GANT61 degradation. In parallel, a new singlet peak is formed at 4.04 ppm, which was attributed to the Ph-CH_2_-NH protons of the newly formed hydrolysis product GANT61-D. Notably, the chemical shift of the new peak from GANT61-D was shown to vary in time as the result of H-bond interactions with the buffer, in agreement with literature data^58^ ^,^ ^59^. Based on NMR data acquired up to 48 h of incubation, a kinetic plot was built to show the hydrolysis of GANT61 (Figure 2(B)).

The data shown in Figure 2(B) clearly highlights the fast kinetics of GANT61 hydrolysis. After 6 h, around 70% of the initial product is hydrolysed into the corresponding GANT61-D, while the plateau is reached at 24 h when around 90% of initial GANT61 is hydrolysed to GANT61-D. From this time point, the reaction is at the equilibrium and no further increase in the relative abundance of GANT61-D is observed up to 48 h incubation.

### Chemical stability of GANT61 checked by HPLC

For the HPLC monitoring of the chemical stability of GANT61, we chose to resort to HILIC, which is nowadays accepted as a common separation mode^60–62^, essentially dedicated to the separation of very polar compounds, such as carbohydrates, amino acids, oligonucleotides, and highly polar natural products. One of the major advantages of HILIC is the easy coupling with MS which extends its applicability to impurity detection^63^. In fact, the use of a low aqueous/high acetonitrile mobile phase significantly improves detection sensitivity for compounds analysed by liquid chromatography (LC)/ESI-MS, thus overcoming the mismatch between normal-phase LC and ESI-MS^64^. Also, ELSD has been widely used in the detection of chromophores-lacking compounds, and thus it proved to be particularly suitable to HILIC applications.

**Figure 3.**
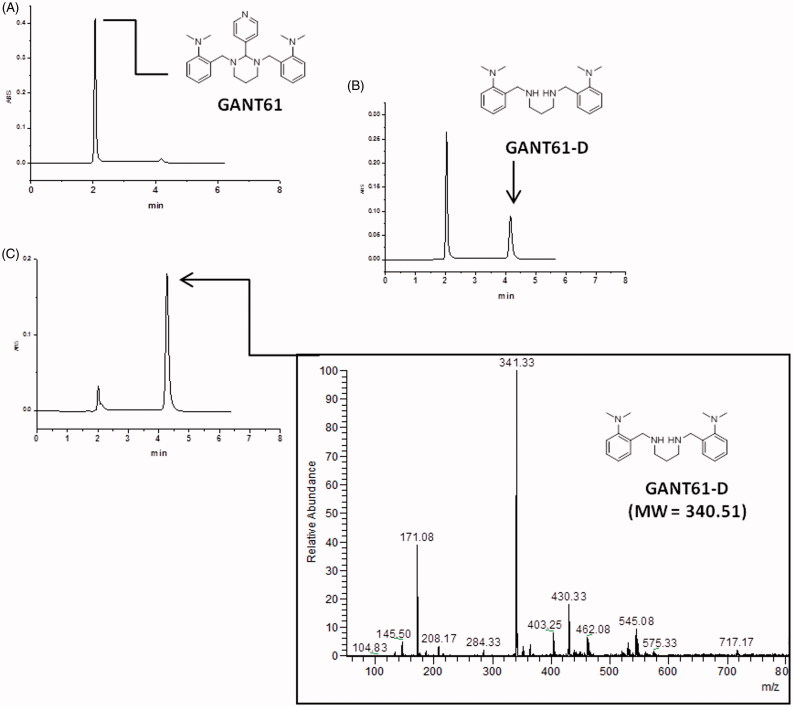
HPLC-UV chromatograms acquired during the time showing the disappearance of GANT61 and the formation of GANT61-D. Sample: GANT61 in CH_3_CN/100 mM NH_4_OAc (pH 4.5) = 95/5 (v/v) (1 mg/ml), column: Acclaim HILIC-10, 3 μm (150 × 4.6 mm I.D.), mobile phase: CH_3_CN/100 mM NH_4_OAc (pH 4.5) = 95/5 (v/v), flow-rate: 1.0 ml/min, detection: UV at 254 nm. (A) t = 0 min; (B) t = 30 min; (C) t = 24 h. The ESI-MS (pos.) spectrum of GANT61-D is included in the box for unequivocal identification.

**Figure 4.**
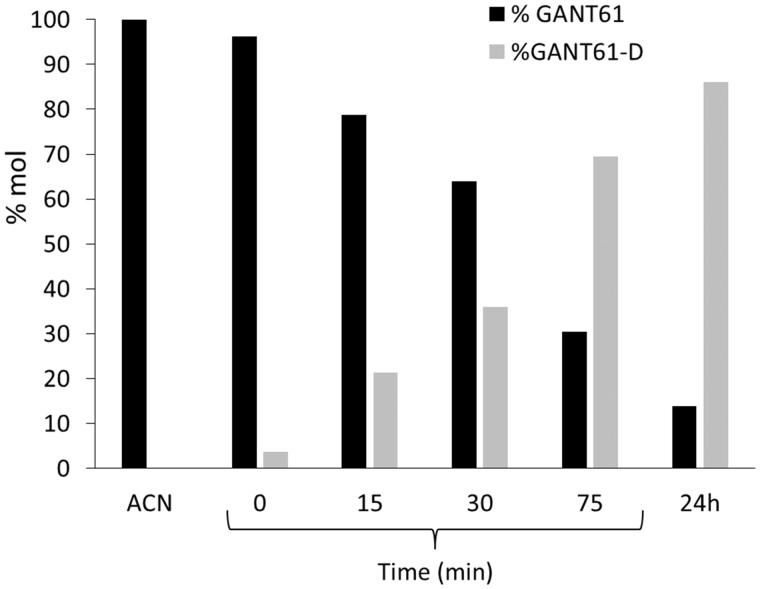
Chemical stability of GANT61 monitored by HPLC as a function of time. The acetonitrile (ACN) solution has been plotted on the left as a control.

After an initial step of investigation for the most suitable chromatographic column operating in the HILIC mode, chromatographic studies were performed on the commercially available Acclaim HILIC-10, 3 µm (150 × 4.6 mm I.D.) column, which was operated under different combinations of aqueous/organic mobile phases. The best mobile phase, consisting of acetonitrile/ammonium acetate 100 mM (pH 4.5) = 95:5 (v/v), yielded the GANT61 peak at about 2 min of retention time (see Figure 3 and Supporting Information Figure S1 for HPLC-UV traces and Supporting Information Figure S2 for the corresponding ELSD chromatograms). When the sample was dissolved in pure acetonitrile (1 mg/ml), the GANT61 peak was the only peak detected for at least 1 h, but when we dissolved GANT61 in the mobile phase (at the same above-mentioned concentration), a new peak attributed to GANT61-D was immediately detected (*t* = 0 in Figure 4) with a retention time equal to 4.20 min. Such a peak proved to increase during the time, to become the main peak after 24 h (Figure 3(C)).

The identity of the two peaks was confirmed by flow injection analysis into an ESI-MS of the above-mentioned solutions (positive ions mode). In particular, the injection of the acetonitrile solution yielded a peak at *m*/*z* = 430.33 (monoisotopic mass), corresponding to the protonated form of GANT61 (MW = 429.61), i.e. [M + H]^+^, while replicated analyses of the GANT61 solution in mobile phase gave a signal at *m*/*z* = 341.33, which was attributed to the mono-protonated form of GANT61-D (MW = 340.51), i.e. [M + H]^+^.

By plotting the HPLC-UV areas measured from the chromatograms acquired during the monitoring against the time (Figure 4) it was pointed out that the GANT61 molecule undergoes the same hydrolytic dissociation described above by NMR analysis, although at lower pH values and hence more rapidly. However, similar to the NMR study, about 90% of GANT61 is converted in GANT61-D after 24 h.

Taken together, NMR and HILIC experiments clearly suggest that GANT61 hydrolyses quickly into GANT61-D, this latter becoming the most abundant species – and hence the bioactive form of GANT61 – in physiological conditions and for incubation time equal or higher than 6 h. Notably, at 24 h, around 90% of GANT61 is converted in GANT61-D.

### Molecular modelling

**Figure 5.**
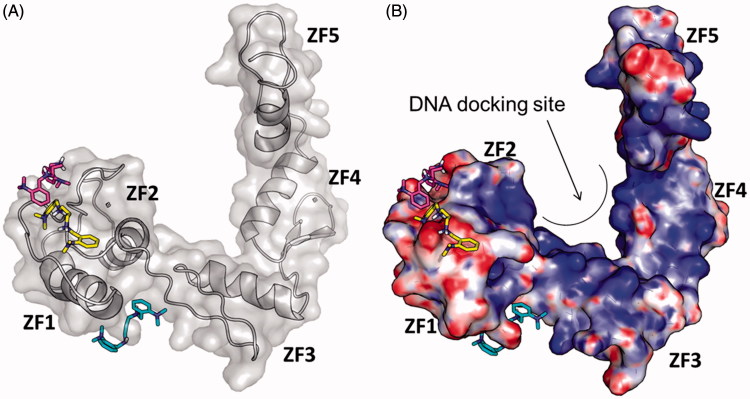
Predicted binding mode of GANT61-D in its three different protonation states: neutral (cyan sticks), mono-protonated (magenta sticks) and di-protonated (yellow sticks). The Gli1ZF crystallographic structure is showed as (A) cartoon and grey transparent surface and (B) surface coloured according to the electrostatic potential calculated by APBS. Red = negatively charged surface; blue = positively charged surface. The positively charged DNA docking site on Gli1ZF is highlighted.

In order to check whether the bioactive form of GANT61, namely GANT61-D, could bind the Gli1ZF, we performed structure-based molecular modelling studies. It is worth mentioning that a similar computational study has been performed earlier by Agyeman et al.^38^ by means of molecular docking simulations on the neutral form of GANT61-D. Different from this prior work, p*K*_a_ predictions clearly indicated that the secondary amino group of GANT61-D is protonated under physiological conditions, with a prevalence of the di-protonated form over the mono-protonated form of the molecule in the pH range 7–7.5^55^. Therefore, to explore the possible binding mode of GANT61-D to Gli1ZF in a physiological context and to compare our results with previous findings, we performed molecular docking of GANT61-D in its neutral, mono- and di-protonated forms by using the same computational settings described by Agyeman et al.^38^ The lowest energy pose of the most populated cluster of each GANT61-D form was visually inspected and used for theoretical affinity studies (see below). Notably, the neutral form of GANT61-D was docked within the groove between ZF2 and ZF3 (Figure 5(A)) in H-bond contacts to Glu250 and Glu298, in excellent agreement with the results published previously (see also the Supporting Information Figure S3)^38^. For the sake of clarity, here we use the numbering scheme of the full-length Gli1 (Glu250 and Glu298 used in this work correspond to Glu119 and Glu167 of the X-ray structure, respectively)^40^. In contrast, protonated forms of GANT61-D were docked preferentially within ZF1 and ZF2 (Figure 5(A)) in a negatively charged surface region of Gli1ZF (Figure 5(B)). Accordingly, we hypothesised that the binding of GANT61-D to Gli1ZF in the most abundant protonation forms is mainly driven by electrostatic forces (detailed binding modes of GANT61-D are showed in Supporting Information Figure S3). Based on molecular docking, GANT61-D is not able to bind within the DNA-binding site of Gli1 that is located within ZF4 and ZF5 as highlighted by X-ray and computational studies (Figure 5(B))^40^. Therefore, it is unlikely that GANT61-D may directly interfere with Gli1 binding to DNA, as instead observed for the direct Gli1/DNA interaction inhibitor GlaB^8^, although we cannot rule out indirect mechanisms leading to alteration in DNA interaction.

Finally, the theoretical affinity of the three protonation forms of GANT61-D towards Gli1ZF was predicted by means of the MM-GBSA method and the XSCORE function^53^ ^,^ ^54^, upon energy minimisation of docking-based binding complexes in explicit water solvent. Results reported in Table 1 unequivocally show that the di-protonated form of GANT61-D holds the strongest affinity for Gli1ZF, whereas the neutral form was predicted to be the lowest affinity species. These results further support the hypothesis of an electrostatic-driven binding of GANT61-D to Gli1ZF that may target the surface region within ZF1 and ZF2 of Gli1ZF, as well as that GANT61-D binds with the highest affinity as protonated species compared to the neutral form. Finally, a good correlation between p*K*_d_ values predicted by XSCORE and experimental affinity measured previously was found^38^.

**Table 1.** Theoretical affinity of GANT61-D to Gli1ZF.

Protonation form of GANT61-D	ΔG binding MM-GBSA (kcal/mol)	XSCORE (p*K*_d_)
Neutral	−25.982 ± 0.008	4.50
Mono-protonated	−28.217 ± 0.006	4.60
Di-protonated	−31.972 ± 0.002	4.65

### Biological activity

**Figure 6.**
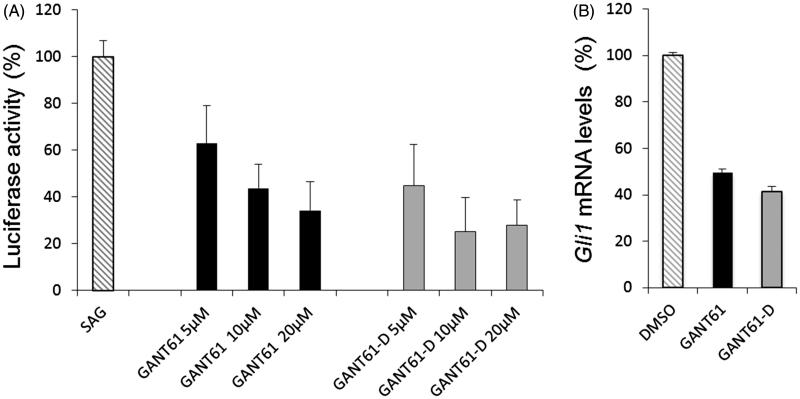
(A) Luciferase reporter assay in NIH3T3 Shh-Light II cells, which shows the dose-dependent inhibition of Hh signalling by GANT61 and GANT61-D after 48 h of treatment. (B) SuFu^−/−^ MEFs were treated for 24 h with GANT61 or GANT61-D (10 μM) or DMSO as a control. *Gli1* mRNA levels were determined by qRT-PCR normalised to β2-microglobulin and *HPRT* expression.

To investigate whether GANT61-D carried the ability to inhibit the Hh signalling, we performed a luciferase functional assay and compared the Hh inhibitory activity of GANT61 with that exerted by GANT61-D. To this end, we treated NIH3T3 Shh-Light II cells, which stably incorporate a Gli-RE^65^, with the synthetic Smo agonist SAG^66^ alone or in combination with different concentrations of the tested compounds. As shown in Figure 6(A), GANT61-D antagonised the SAG-induced Hh pathway in a dose-dependent manner and with comparable efficacy as the parent GANT61. Next, we analysed the effect of GANT61-D in SuFu^−/−^ MEFs, in which the constitutive activation of the pathway is a consequence of the deletion of the negative regulator *SuFu* gene. GANT61-D at 10 µM concentration significantly reduced the expression of Gli1, the final and most powerful effector of the Hh signalling (Figure 6(B)), already after 24 h, in agreement with the concept that the bioactive form of GANT61 to mediate Hh inhibition is its diamine derivative GANT61-D.

## Discussion

The Hh signalling pathway is involved in many different types of cancer and its inhibition by small molecules is nowadays considered an effective anticancer strategy^4^ ^,^ ^6^ ^,^ ^65^. Although the initial enthusiasm for the approval by the FDA of two antagonists of the Smo receptor^25^ ^,^ ^26^, drug-resistant Smo mutations and aberrant activation of Hh signalling downstream of Smo have been identified in clinical patients, which pointed to the serious need of alternative approaches^29–33^. Besides Smo, one of the most profitable Hh targets is Gli1, the final and most powerful effector of Hh signalling^6^ ^,^ ^8^. GANT61 has been identified as the first small molecule inhibitor of Gli1 and to date, it is used as a reference control in multiple studies focusing on Hh signalling transduction and modulation by small molecules^35^. The chemical instability of GANT61 was first highlighted in 2010 by Lauth et al.^36^ ^,^ ^37^, and further addressed in 2014 by Agyeman et al.^38^. Besides these reports, here we aim to provide additional insights into the mechanism and kinetics of GANT61 hydrolysis and to complement the knowledge on the mechanism of actions of this reference Hh signalling inhibitor. To these aims, GANT61 and its diamine derivative GANT61-D were obtained by chemical synthesis, while the kinetics of GANT61 hydrolysis was monitored by high-resolution analytical tools such as NMR spectroscopy and HILIC. Results of our study show that GANT61 hydrolysed to the diamine GANT61-D and the aldehyde GANT61-A. This process is endowed with a rather fast kinetics, as a plateau is reached in 24 h when about 90% of GANT61 is converted in GANT61-D. These results unequivocally indicate that the bioactive form of GANT61 in cell-based experiments is the diamine derivative GANT61-D, particularly when incubation time is equal or higher than 6 h.

To corroborate this hypothesis, and to further support NMR and HILIC data, we performed molecular modelling and functional studies. Multiple protonation states of GANT61-D were considered in molecular docking simulations against Gli1ZF, including the neutral form that – although being endowed with a very low probability to exist in physiological conditions – has been the subject of a recent modelling study^38^. Results of the present computational analysis show that the protonated forms are most affine to Gli1ZF compared to the neutral form, and bind in a negatively charged region of the protein in correspondence of ZF1 and ZF2 thus suggesting that electrostatic forces may be crucial for intermolecular recognition and binding.

Finally, the Hh inhibitory properties of GANT61 and GANT61-D were evaluated in NIH3T3 Shh-Light II cells, which stably incorporate a Gli-RE, and in SuFu^−/−^ MEFs, in which the pathway is constitutively active. In both assays, GANT61-D showed its ability to antagonise Hh signalling and to inhibit Gli1 expression with comparable efficacy as GANT61. Our findings strongly support that GANT61-D is the bioactive form of GANT61 able to counteract the Hh signalling at downstream level, thus limiting the oncogenic potential of the pathway occurring in a context of Smo-independent or Gli1 hyperactivation by alternative mechanisms.

## Conclusion

In this work, we shed light on the mechanism and kinetics of GANT61 hydrolysis, and further complement previous works in substantiating that GANT61-D is the bioactive form of GANT61 in Hh inhibition. Molecular modelling studies identified the main pharmacophores of GANT61-D that are relevant for the binding to Gli1ZF and suggested some strategies to generate optimised derivatives. For example, molecular rigidification and the design of GANT61-D analogues with constrained diamine linker may be undertaken to limit the conformational freedom of the molecule; phenyl rings may be decorated with functional groups that improve the interaction with Gli1ZF, or with chemical moieties that may ameliorate the pharmacokinetics profile of the lead; the aryl-dimethylamino group may be also replaced by different chemotypes, as it seems not particularly relevant for the interaction with Gli1ZF that we found to be driven mostly by electrostatic forces. Besides these suggestions, we hope that results of our integrated and multidisciplinary effort will inspire additional routes to design more potent and effective Hh inhibitors based on the scaffold of GANT61-D.

## Supplementary Material

IENZ_1419221_Supplementary_Material.pdf

## References

[1] Masimirembwa CM, Bredberg U, Andersson TB. Metabolic stability for drug discovery and development: pharmacokinetic and biochemical challenges. Clin Pharmacokinet 2003;42:515–28. 12793837 10.2165/00003088-200342060-00002

[2] Shu YZ, Johnson BM, Yang TJ. Role of biotransformation studies in minimizing metabolism-related liabilities in drug discovery. AAPS J 2008;10:178–92. 18446518 10.1208/s12248-008-9016-9 PMC2751461

[3] Varjosalo M, Taipale J. Hedgehog: functions and mechanisms. Genes Dev 2008;22:2454–72. 18794343 10.1101/gad.1693608

[4] Ng JM, Curran T. The Hedgehog’s tale: developing strategies for targeting cancer. Nat Rev Cancer 2011;11:493–501. 21614026 10.1038/nrc3079 PMC3576812

[5] Iovine V, Mori M, Calcaterra A, et al One hundred faces of cyclopamine. Curr Pharm Des 2016;22:1658–81. 26759083 10.2174/1381612822666160112130157

[6] Infante P, Alfonsi R, Botta B, et al Targeting GLI factors to inhibit the Hedgehog pathway. Trends Pharmacol Sci 2015;36:547–58. 26072120 10.1016/j.tips.2015.05.006

[7] Infante P, Alfonsi R, Ingallina C, et al Inhibition of Hedgehog-dependent tumors and cancer stem cells by a newly identified naturally occurring chemotype. Cell Death Dis 2016;7:e2376. 27899820 10.1038/cddis.2016.195 PMC5059851

[8] Infante P, Mori M, Alfonsi R, et al Gli1/DNA interaction is a druggable target for Hedgehog-dependent tumors. EMBO J 2015;34:200–17. 25476449 10.15252/embj.201489213 PMC4298015

[9] Berman DM, Karhadkar SS, Hallahan AR, et al Medulloblastoma growth inhibition by hedgehog pathway blockade. Science 2002;297:1559–61. 12202832 10.1126/science.1073733

[10] De Smaele E, Fragomeli C, Ferretti E, et al An integrated approach identifies Nhlh1 and Insm1 as Sonic Hedgehog-regulated genes in developing cerebellum and medulloblastoma. Neoplasia 2008;10:89–98. 18231642 10.1593/neo.07891 PMC2213903

[11] Di Magno L, Manzi D, D’Amico D, et al Druggable glycolytic requirement for Hedgehog-dependent neuronal and medulloblastoma growth. Cell Cycle 2014;13:3404–13. 25485584 10.4161/15384101.2014.952973 PMC4613849

[12] Ridzewski R, Rettberg D, Dittmann K, et al Hedgehog inhibitors in rhabdomyosarcoma: a comparison of four compounds and responsiveness of four cell lines. Front Oncol 2015;5:130. 26106586 10.3389/fonc.2015.00130 PMC4459089

[13] Oue T, Uehara S, Yamanaka H, et al Hedgehog signal inhibitors suppress the invasion of human rhabdomyosarcoma cells. Pediatr Surg Int 2013;29:1153–8. 23989521 10.1007/s00383-013-3369-6 PMC3824305

[14] Feldmann G, Dhara S, Fendrich V, et al Blockade of hedgehog signaling inhibits pancreatic cancer invasion and metastases: a new paradigm for combination therapy in solid cancers. Cancer Res 2007;67:2187–96. 17332349 10.1158/0008-5472.CAN-06-3281 PMC3073370

[15] Varnat F, Duquet A, Malerba M, et al Human colon cancer epithelial cells harbour active HEDGEHOG-GLI signalling that is essential for tumour growth, recurrence, metastasis and stem cell survival and expansion. EMBO Mol Med 2009;1:338–51. 20049737 10.1002/emmm.200900039 PMC3378144

[16] Karhadkar SS, Bova GS, Abdallah N, et al Hedgehog signalling in prostate regeneration, neoplasia and metastasis. Nature 2004;431:707–12. 15361885 10.1038/nature02962

[17] Watkins DN, Berman DM, Burkholder SG, et al Hedgehog signalling within airway epithelial progenitors and in small-cell lung cancer. Nature 2003;422:313–17 12629553 10.1038/nature01493

[18] Clement V, Sanchez P, de Tribolet N, et al HEDGEHOG-GLI1 signaling regulates human glioma growth, cancer stem cell self-renewal, and tumorigenicity. Curr Biol 2007;17:165–172 17196391 10.1016/j.cub.2006.11.033 PMC1855204

[19] Zhao C, Chen A, Jamieson CH, et al Hedgehog signalling is essential for maintenance of cancer stem cells in myeloid leukaemia. Nature 2009;458:776–9. 19169242 10.1038/nature07737 PMC2946231

[20] Reya T, Morrison SJ, Clarke MF, Weissman IL. Stem cells, cancer, and cancer stem cells. Nature 2001;414:105–11. 11689955 10.1038/35102167

[21] Pardal R, Clarke MF, Morrison SJ. Applying the principles of stem-cell biology to cancer. Nat Rev Cancer 2003;3:895–902. 14737120 10.1038/nrc1232

[22] Coni S, Infante P, Gulino A. Control of stem cells and cancer stem cells by Hedgehog signaling: pharmacologic clues from pathway dissection. Biochem Pharmacol 2013;85:623–8. 23148911 10.1016/j.bcp.2012.11.001

[23] Chen K, Huang YH, Chen JL. Understanding and targeting cancer stem cells: therapeutic implications and challenges. Acta Pharmacol Sin 2013;34:732–40. 23685952 10.1038/aps.2013.27 PMC3674516

[24] Ismail F, Winkler DA. Getting to the source: selective drug targeting of cancer stem cells. ChemMedChem 2014;9:885–98. 24760779 10.1002/cmdc.201400068

[25] Dlugosz A, Agrawal S, Kirkpatrick P. Vismodegib. Nat Rev Drug Discov 2012;11:437–8. 22653209 10.1038/nrd3753 PMC3383648

[26] Mullard A. 2015 FDA drug approvals. Nat Rev Drug Discov 2016;15:73–6. 26837582 10.1038/nrd.2016.15

[27] Ruat M, Hoch L, Faure H, Rognan D. Targeting of smoothened for therapeutic gain. Trends Pharmacol Sci 2014;35:237–46. 24703627 10.1016/j.tips.2014.03.002

[28] Rimkus TK, Carpenter RL, Qasem S, et al Targeting the sonic hedgehog signaling pathway: review of smoothened and GLI inhibitors. Cancers (Basel) 2016;8:22. 10.3390/cancers8020022 PMC4773745 26891329

[29] Yauch RL, Dijkgraaf GJ, Alicke B, et al Smoothened mutation confers resistance to a Hedgehog pathway inhibitor in medulloblastoma. Science 2009;326:572–4. 19726788 10.1126/science.1179386 PMC5310713

[30] Atwood SX, Sarin KY, Whitson RJ, et al Smoothened variants explain the majority of drug resistance in basal cell carcinoma. Cancer Cell 2015;27:342–53. 25759020 10.1016/j.ccell.2015.02.002 PMC4357167

[31] Sharpe HJ, Pau G, Dijkgraaf GJ, et al Genomic analysis of smoothened inhibitor resistance in basal cell carcinoma. Cancer Cell 2015;27:327. 25759019 10.1016/j.ccell.2015.02.001 PMC5675004

[32] Buonamici S, Williams J, Morrissey M, et al Interfering with resistance to smoothened antagonists by inhibition of the PI3K pathway in medulloblastoma. Sci Transl Med 2010;2:51ra70. 10.1126/scitranslmed.3001599 PMC3422576 20881279

[33] Metcalfe C, de Sauvage FJ. Hedgehog fights back: mechanisms of acquired resistance against smoothened antagonists. Cancer Res 2011;71:5057–61. 21771911 10.1158/0008-5472.CAN-11-0923

[34] Gonnissen A, Isebaert S, Haustermans K. Targeting the Hedgehog signaling pathway in cancer: beyond smoothened. Oncotarget 2015;6:13899–913. 26053182 10.18632/oncotarget.4224 PMC4546439

[35] Lauth M, Bergström A, Shimokawa T, Toftgård R. Inhibition of GLI-mediated transcription and tumor cell growth by small-molecule antagonists. Proc Natl Acad Sci USA 2007;104:8455–60. 17494766 10.1073/pnas.0609699104 PMC1866313

[36] Lauth M, Rohnalter V, Bergstrom A, et al Correction to “Antipsychotic drugs regulate hedgehog signaling by modulation of 7-dehydrocholesterol reductase levels”. Mol Pharmacol 2011;79:793. 10.1124/mol.110.066431 20558592

[37] Lauth M, Rohnalter V, Bergström A, et al Antipsychotic drugs regulate hedgehog signaling by modulation of 7-dehydrocholesterol reductase levels. Mol Pharmacol 2010;78:486–96. 20558592 10.1124/mol.110.066431

[38] Agyeman A, Jha BK, Mazumdar T, Houghton JA. Mode and specificity of binding of the small molecule GANT61 to GLI determines inhibition of GLI-DNA binding. Oncotarget 2014;5:4492–503. 24962990 10.18632/oncotarget.2046 PMC4147340

[39] Glasoe PK, Long FA. Use of glass electrodes to measure acidities in deuterium oxide. J Phys Chem 1960;64:188–90.

[40] Pavletich NP, Pabo CO. Crystal structure of a five-finger GLI-DNA complex: new perspectives on zinc fingers. Science 1993;261:1701–7. 8378770 10.1126/science.8378770

[41] Morris GM, Huey R, Lindstrom W, et al AutoDock4 and AutoDockTools4: automated docking with selective receptor flexibility. J Comput Chem 2009;30:2785–91. 19399780 10.1002/jcc.21256 PMC2760638

[42] Cirigliano A, Menta S, Mori M, et al *S. cerevisiae* as a tool to select inhibitors of the deneddylating activity of the COP9 signalosome. Yeast 2015;32:S175

[43] Mori M, Dietrich U, Manetti F, Botta M. Molecular dynamics and DFT study on HIV-1 nucleocapsid protein-7 in complex with viral genome. J Chem Inf Model 2010;50:638–50. 20201584 10.1021/ci100070m

[44] Mori M, Massaro A, Calderone V, et al Discovery of a new class of potent MMP inhibitors by structure-based optimization of the arylsulfonamide scaffold. ACS Med Chem Lett 2013;4:565–9. 24900710 10.1021/ml300446a PMC4027459

[45] Mori M, Moraca F, Deodato D, et al Discovery of the first potent and selective *Mycobacterium tuberculosis* Zmp1 inhibitor. Bioorg Med Chem Lett 2014;24:2508–11. 24767848 10.1016/j.bmcl.2014.04.004

[46] Baker NA, Sept D, Joseph S, et al Electrostatics of nanosystems: application to microtubules and the ribosome. Proc Natl Acad Sci USA 2001;98:10037–41. 11517324 10.1073/pnas.181342398 PMC56910

[47] Case DA, Darden TA, Cheatham TE, et al AMBER 12. San Francisco, CA: University of California; 2012.

[48] Cau Y, Fiorillo A, Mori M, et al Molecular dynamics simulations and structural analysis of Giardia duodenalis 14-3-3 protein-protein interactions. J Chem Inf Model 2015;55:2611–22. 26551337 10.1021/acs.jcim.5b00452

[49] Cau Y, Mori M, Supuran CT, Botta M. Mycobacterial carbonic anhydrase inhibition with phenolic acids and esters: kinetic and computational investigations. Org Biomol Chem 2016;14:8322–30. 27530867 10.1039/c6ob01477a

[50] Mori M, Nucci A, Lang MC, et al Functional and structural characterization of 2-amino-4-phenylthiazole inhibitors of the HIV-1 nucleocapsid protein with antiviral activity. ACS Chem Biol 2014;9:1950–5. 24988251 10.1021/cb500316h

[51] Sholokh M, Improta R, Mori M, et al Tautomers of a fluorescent G surrogate and their distinct photophysics provide additional information channels. Angew Chem Int Ed Engl 2016;55:7974–8. 27273741 10.1002/anie.201601688 PMC4978544

[52] Mori M, Manetti F, Botta M. Predicting the binding mode of known NCp7 inhibitors to facilitate the design of novel modulators. J Chem Inf Model 2011;51:446–54. 21171587 10.1021/ci100393m

[53] Miller BR 3rd, McGee TD Jr, Swails JM, et al MMPBSA.py: an efficient program for end-state free energy calculations. J Chem Theory Comput 2012;8:3314–21. 26605738 10.1021/ct300418h

[54] Wang R, Lai L, Wang S. Further development and validation of empirical scoring functions for structure-based binding affinity prediction. J Comput-Aided Mol Des 2002;16:11–26. 12197663 10.1023/a:1016357811882

[55] Milletti F, Storchi L, Sforna G, Cruciani G. New and original pKa prediction method using grid molecular interaction fields. J Chem Inf Model 2007;47:2172–81. 17910431 10.1021/ci700018y

[56] Chenna V, Hu C, Khan SR. Synthesis and cytotoxicity studies of Hedgehog enzyme inhibitors SANT-1 and GANT-61 as anticancer agents. J Environ Sci Health A Tox Hazard Subst Environ Eng 2014;49:641–7. 24521409 10.1080/10934529.2014.865425

[57] Valensin D, Cau Y, Calandro P, et al Molecular insights to the bioactive form of BV02, a reference inhibitor of 14-3-3sigma protein-protein interactions. Bioorg Med Chem Lett 2016;26:894–8. 26774582 10.1016/j.bmcl.2015.12.066

[58] Mitra A, Seaton PJ, Ali Assarpour R, Williamson T. Unprecedented concentration dependent chemical shift variation in 1H-NMR studies: a caveat in the investigations of molecular recognition and structure elucidation. Tetrahedron 1998;54:15489–98.

[59] D’Acquarica I, Calcaterra A, Sacco F, et al Stereochemical preference of 2′-deoxycytidine for chiral bis(diamido)-bridged basket resorcin[4]arenes. Chirality 2013;25:840–51. 24038320 10.1002/chir.22224

[60] Alpert AJ. Hydrophilic-interaction chromatography for the separation of peptides, nucleic acids and other polar compounds. J Chromatogr 1990;499:177–96. 2324207 10.1016/s0021-9673(00)96972-3

[61] Hemstrom P, Irgum K. Hydrophilic interaction chromatography. J Sep Sci 2006;29:1784–821. 16970185 10.1002/jssc.200600199

[62] Nguyen HP, Schug KA. The advantages of ESI-MS detection in conjunction with HILIC mode separations: fundamentals and applications. J Sep Sci 2008;31:1465–80. 18401862 10.1002/jssc.200700630

[63] Dejaegher B, Vander Heyden Y. HILIC methods in pharmaceutical analysis. J Sep Sci 2010;33:698–715. 20183826 10.1002/jssc.200900742

[64] Lammerhofer M. HILIC and mixed-mode chromatography: the rising stars in separation science. J Sep Sci 2010;33:679–80. 20358633 10.1002/jssc.201090015

[65] Taipale J, Chen JK, Cooper MK, et al Effects of oncogenic mutations in Smoothened and Patched can be reversed by cyclopamine. Nature 2000;406:1005–9. 10984056 10.1038/35023008

[66] Chen JK, Taipale J, Cooper MK, Beachy PA. Inhibition of Hedgehog signaling by direct binding of cyclopamine to smoothened. Genes Dev 2002;16:2743–8. 12414725 10.1101/gad.1025302 PMC187469

