# Supplementary material for: Chemical, computational and functional insights into the chemical stability of the Hedgehog pathway inhibitor GANT61
Source: J Enzyme Inhib Med Chem. 2018 Jan 17;33(1):349–58. doi: 10.1080/14756366.2017.1419221 (PMC6009951; doi:10.1080/14756366.2017.1419221)

Chemical, computational and functional insights into the chemical stability of the Hedgehog pathway inhibitor GANT61

Supporting Information Figures

Figure S1. HPLC-UV chromatograms .... page S2

Figure S2. HPLC-ELSD chromatograms .... page S3

Figure S2. Predicted binding mode of GANT61-D to Gli1ZF.. page S4

NMR and MS Spectra

^{1}H NMR of 2-(dimethylamino)benzaldehyde .... page S5

^{13}C NMR of 2-(dimethylamino)benzaldehyde .... page S6

^{1}H NMR of GANT61-D pageS7

^{13}C NMR of GANT61-D .... page S8

^{1}H NMR of GANT61 .... pages S9-S10

^{13}C NMR of GANT61 .... page S11

ESI MS of GANT61-D .... page S12

ESI MS of GANT61 .... page S13

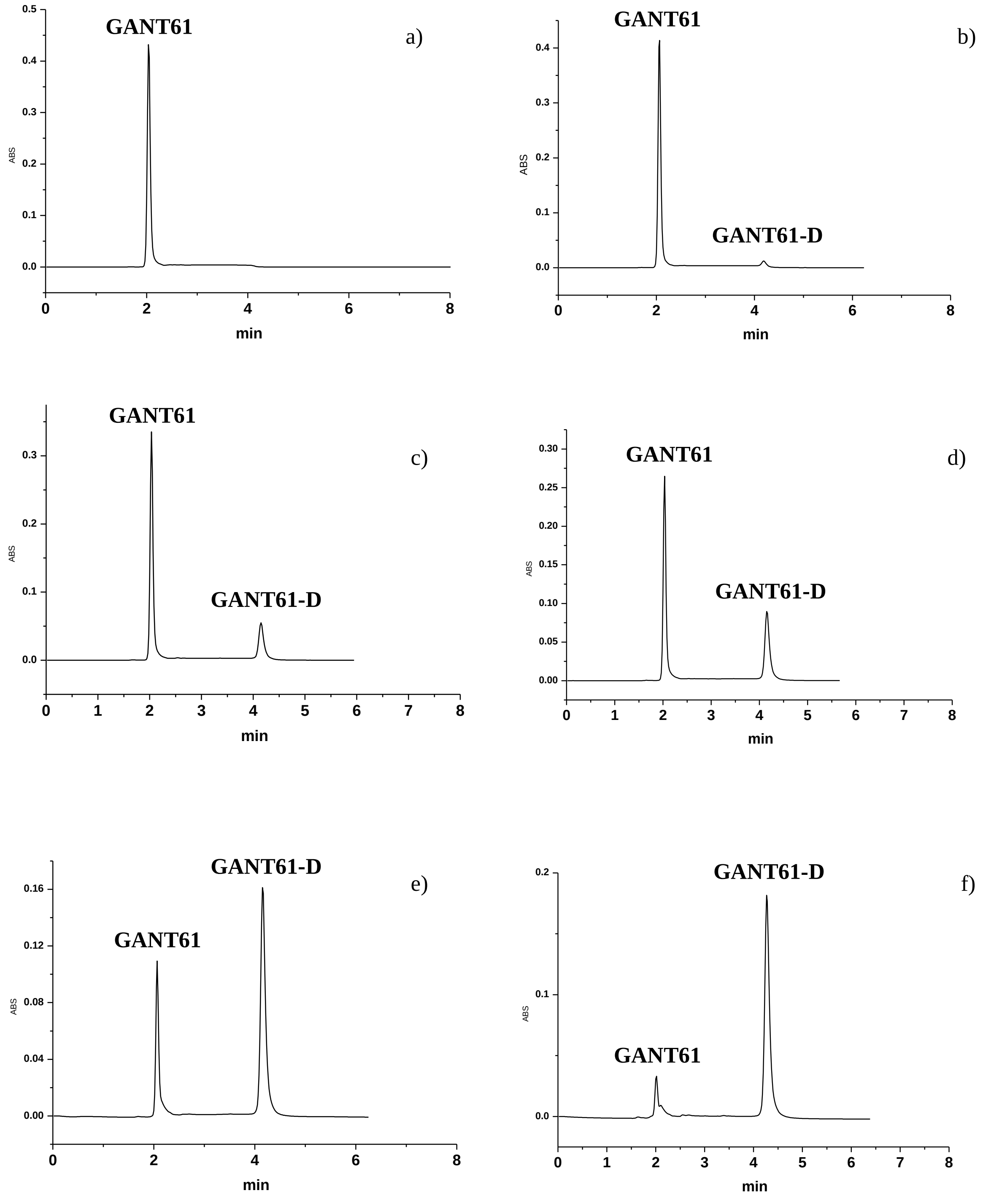

Figure S1. HPLC-UV chromatograms at different times confirm the disappearance of GANT61 and the formation of GANT61-D. **a)** GANT61 in CH_3CN; **b–f)** GANT61 in CH_3CN/100 mM NH_4OAc, pH=4.5; 95/5, v/v: b) t = 0 min; c) t = 15 min; d) t = 30 min; e) t = 75 min; f) t = 24 h.

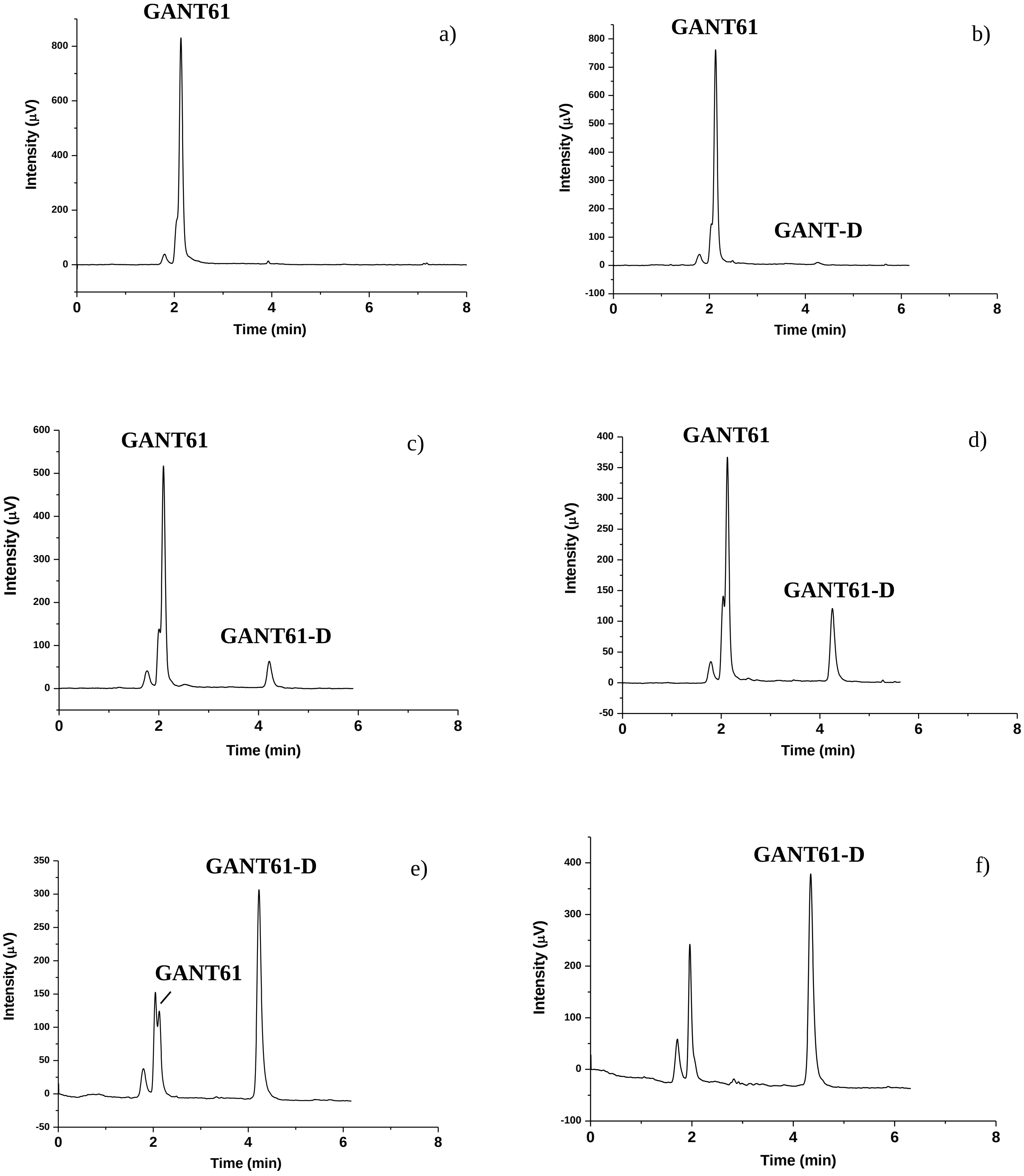

Figure S2. HPLC-ELSD chromatograms at different times confirm the disappearance of GANT61 and the formation of GANT61-D. **a)** GANT61 in CH_3CN; **b–f)** GANT61 in CH_3CN/100 mM NH_4OAc, pH=4.5; 95/5, v/v: b) t = 0 min; c) t = 15 min; d) t = 30 min; e) t = 75 min; f) t = 24 h.

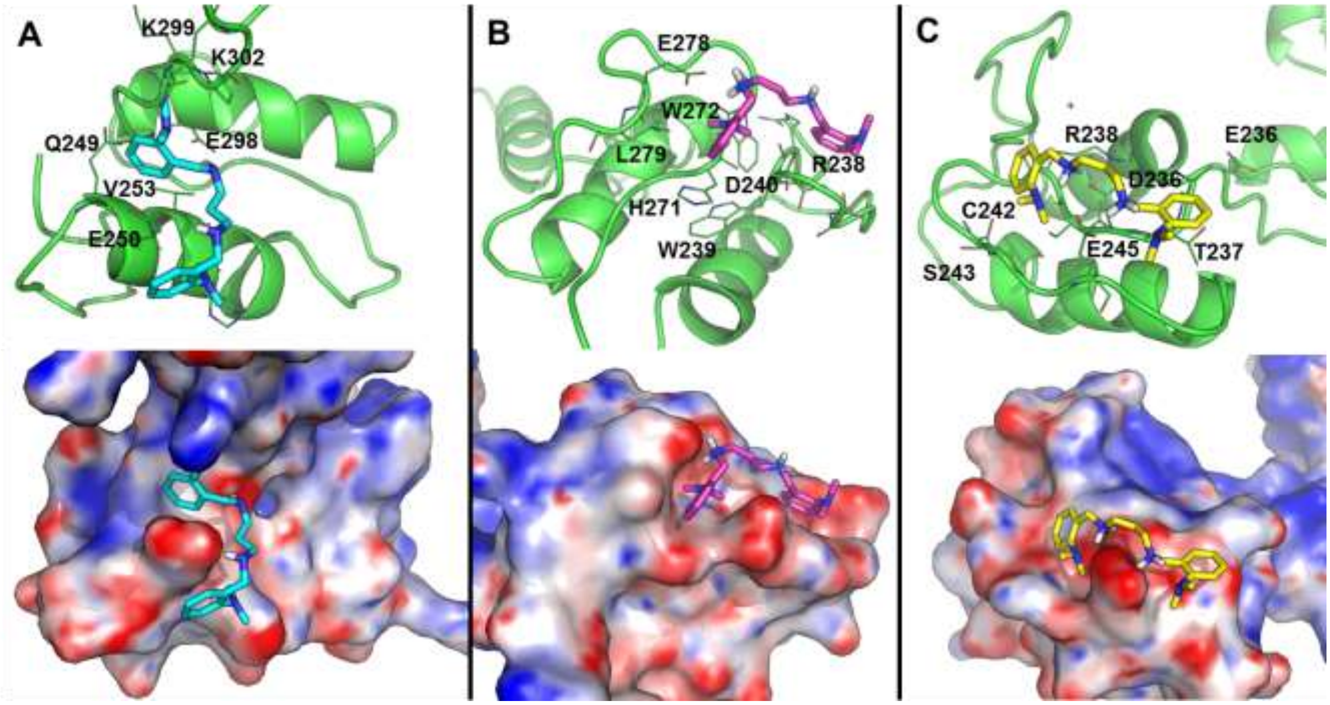

Figure S3. Magnification of GANT61-D binding modes to Gli1ZF. **A)** predicted binding mode of the neutral form of GANT61-D (cyan sticks); **B)** predicted binding mode of the mono-protonated form of GANT61-D (magenta sticks); **C)** predicted binding mode of the di-protonated form of GANT61-D (yellow sticks). Gli1ZF is showed as green cartoon in top panels and surface colored according with electrostatic potential in bottom panels. Residues within 4 Å from GANT61-D are shown as green sticks and are labeled.

^{1}H NMR of 2-(dimethylamino)benzaldehyde

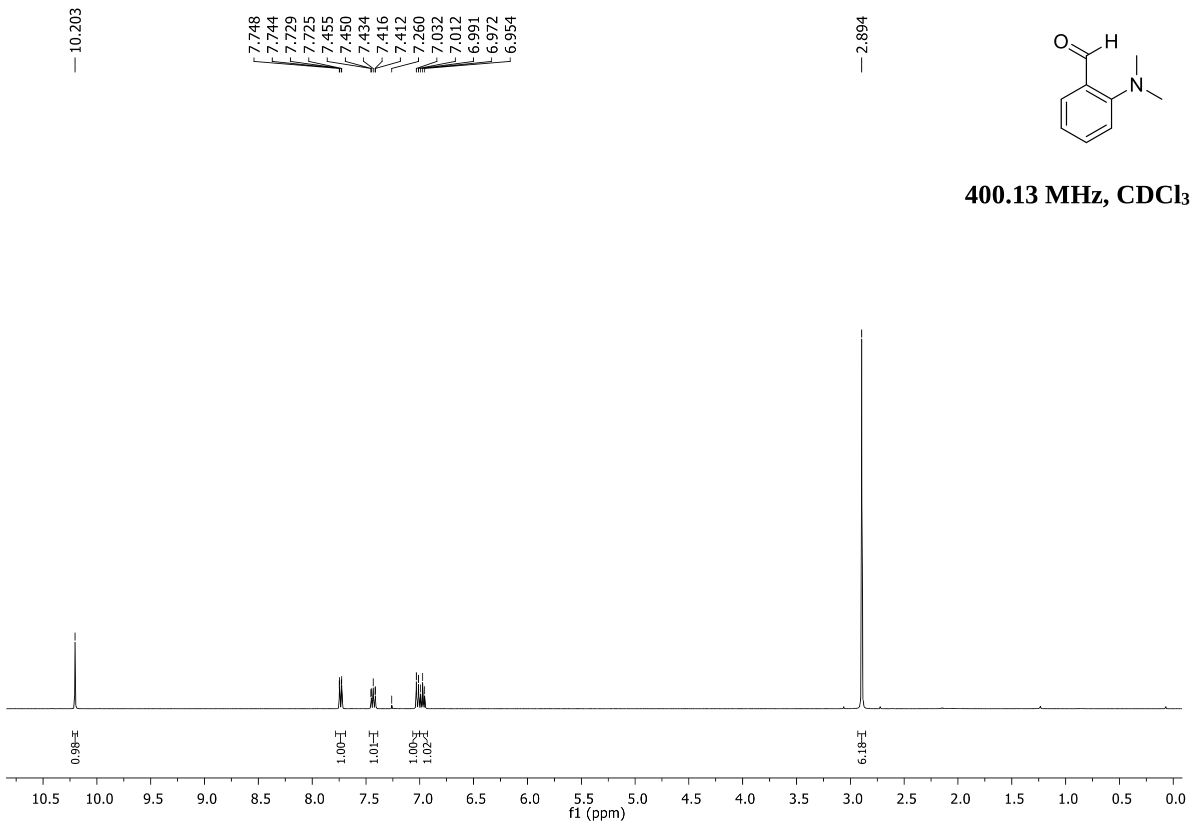

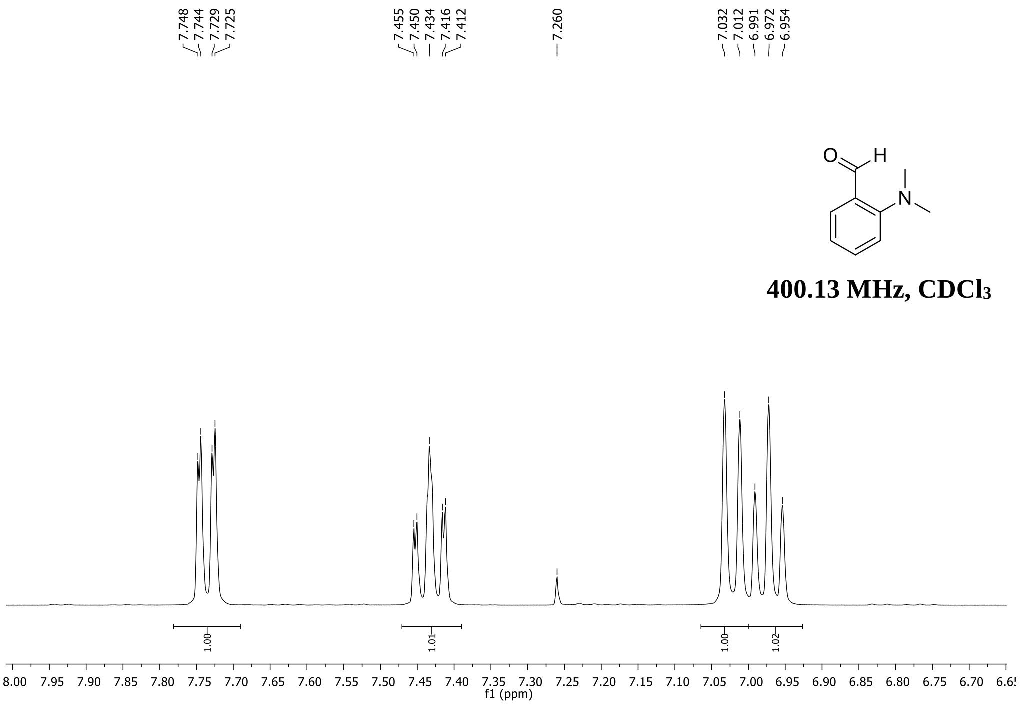

^{13}C NMR of 2-(dimethylamino)benzaldehyde

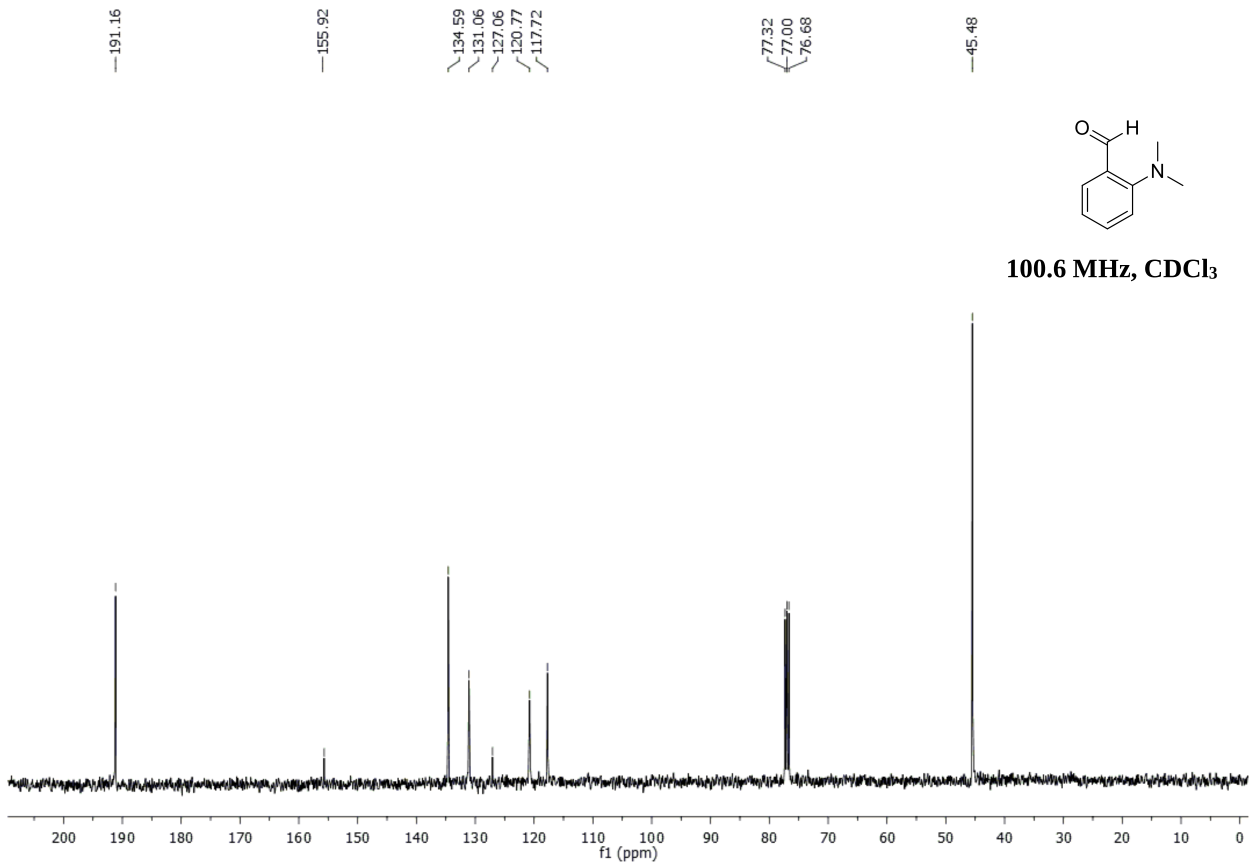

^{1}H NMR of GANT61-D

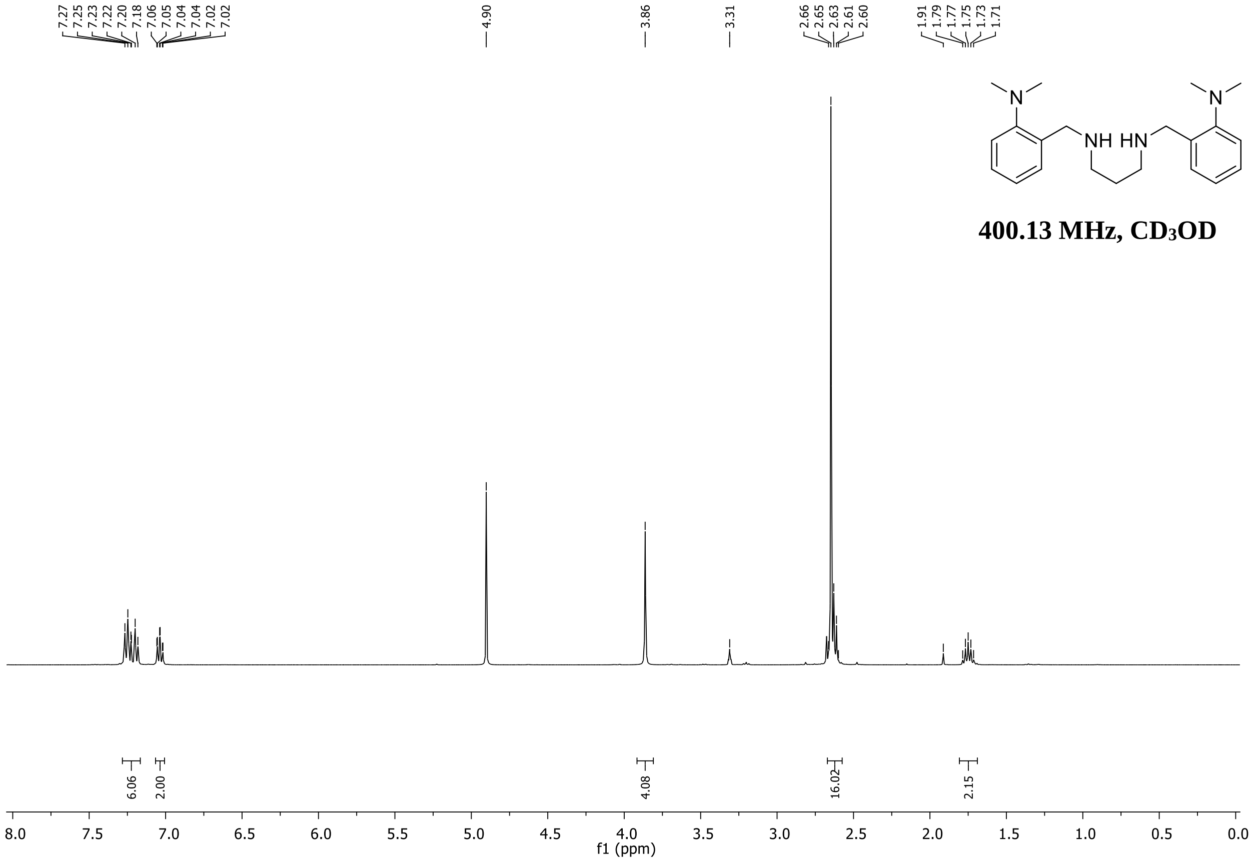

^{13}C NMR of GANT61-D

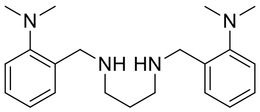

100.6 MHz, CD$_3$OD

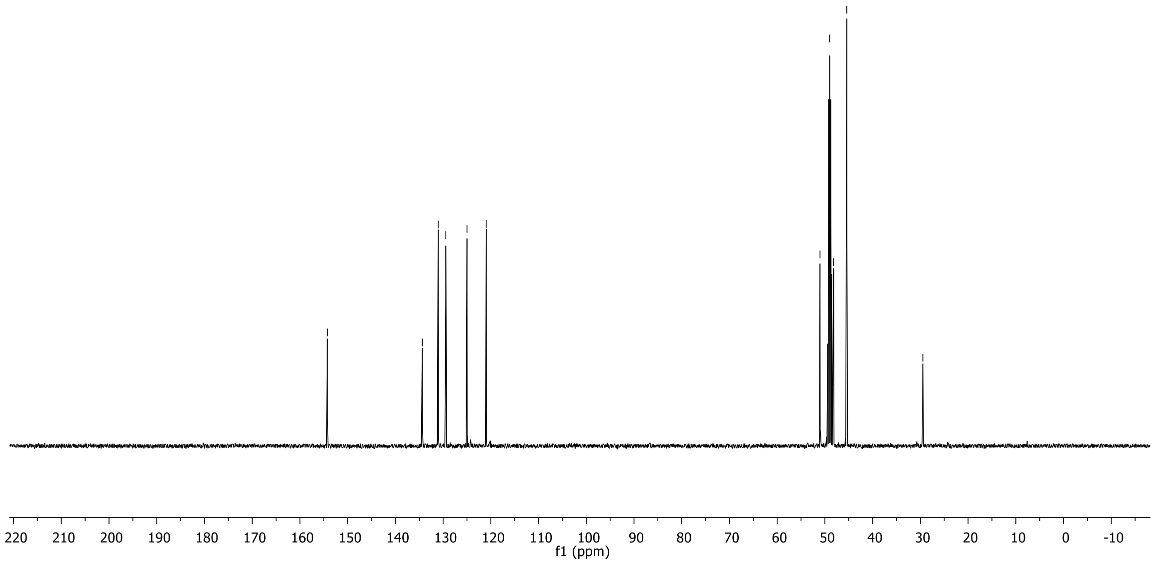

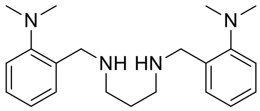

100.6 MHz, CD$_3$OD

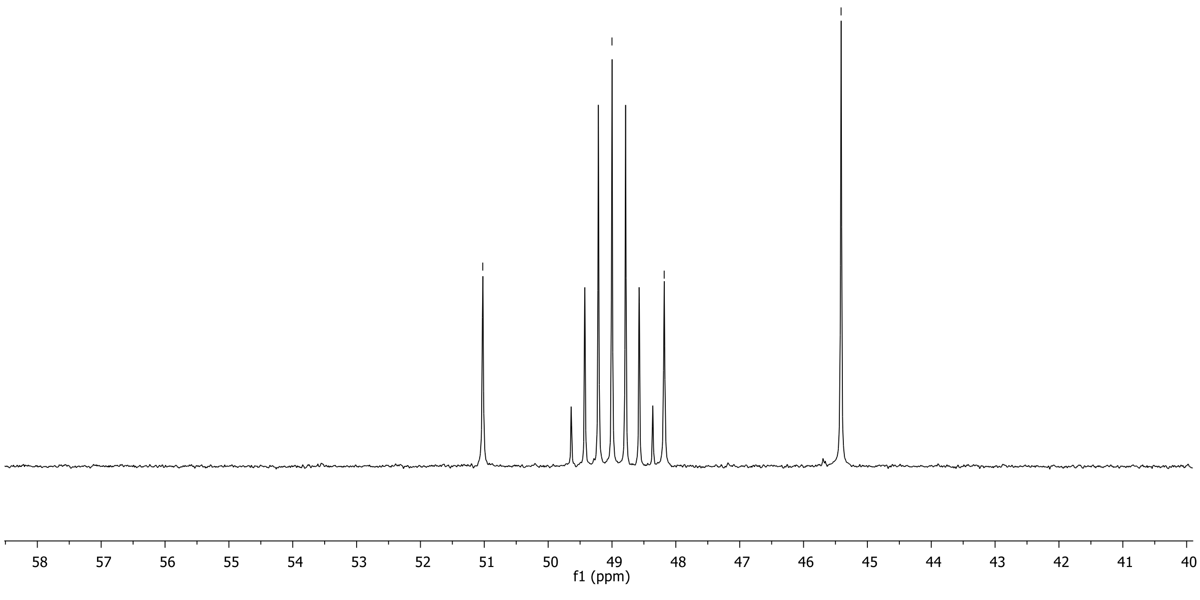

^{1}H NMR of GANT61

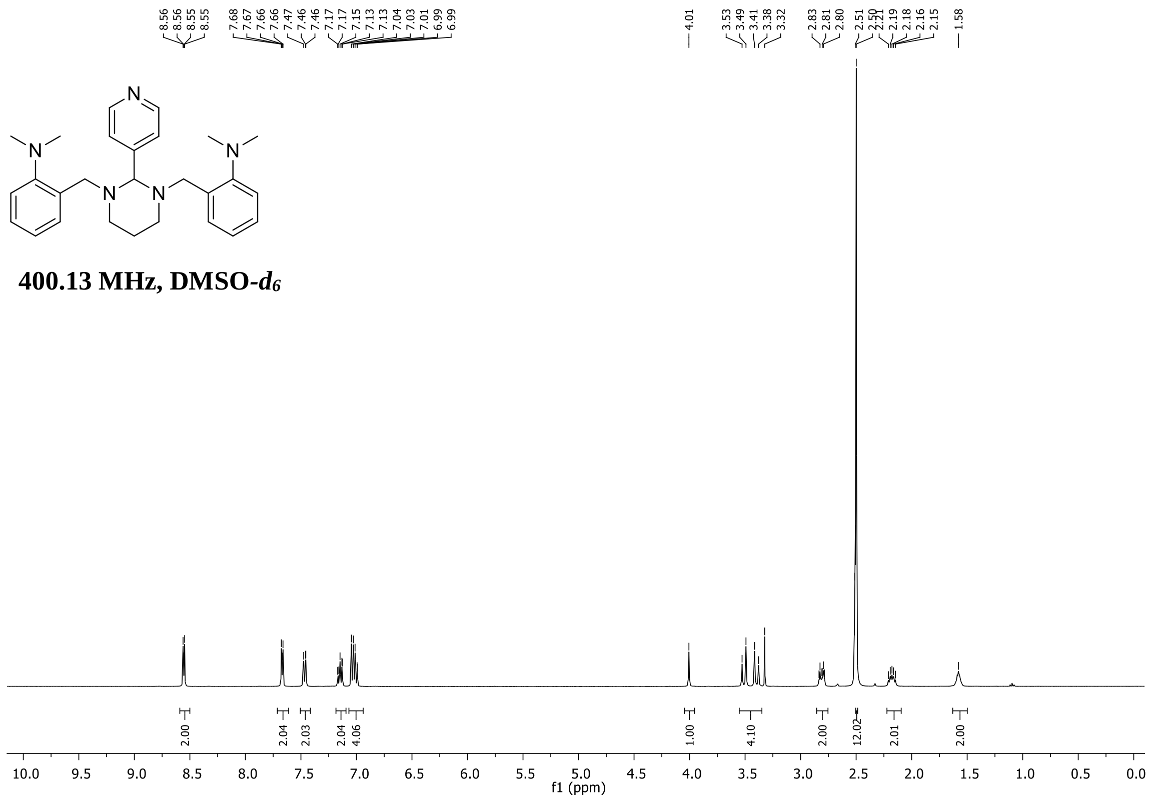

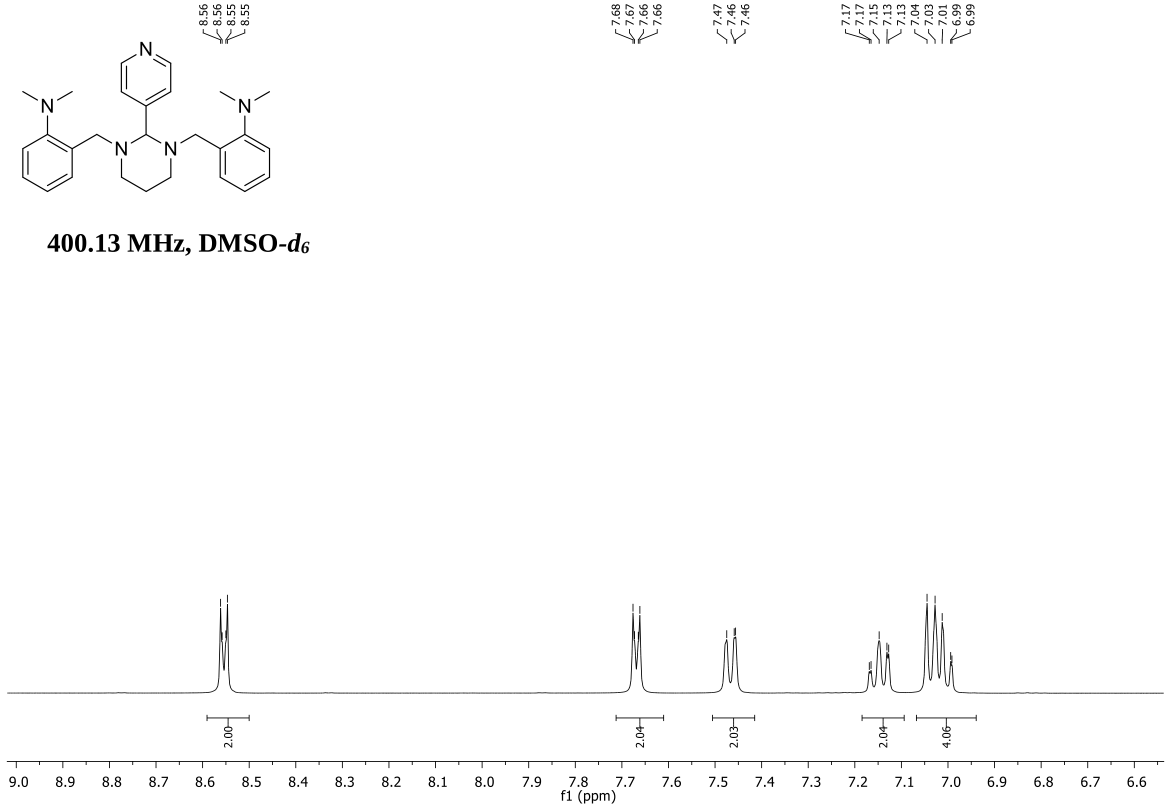

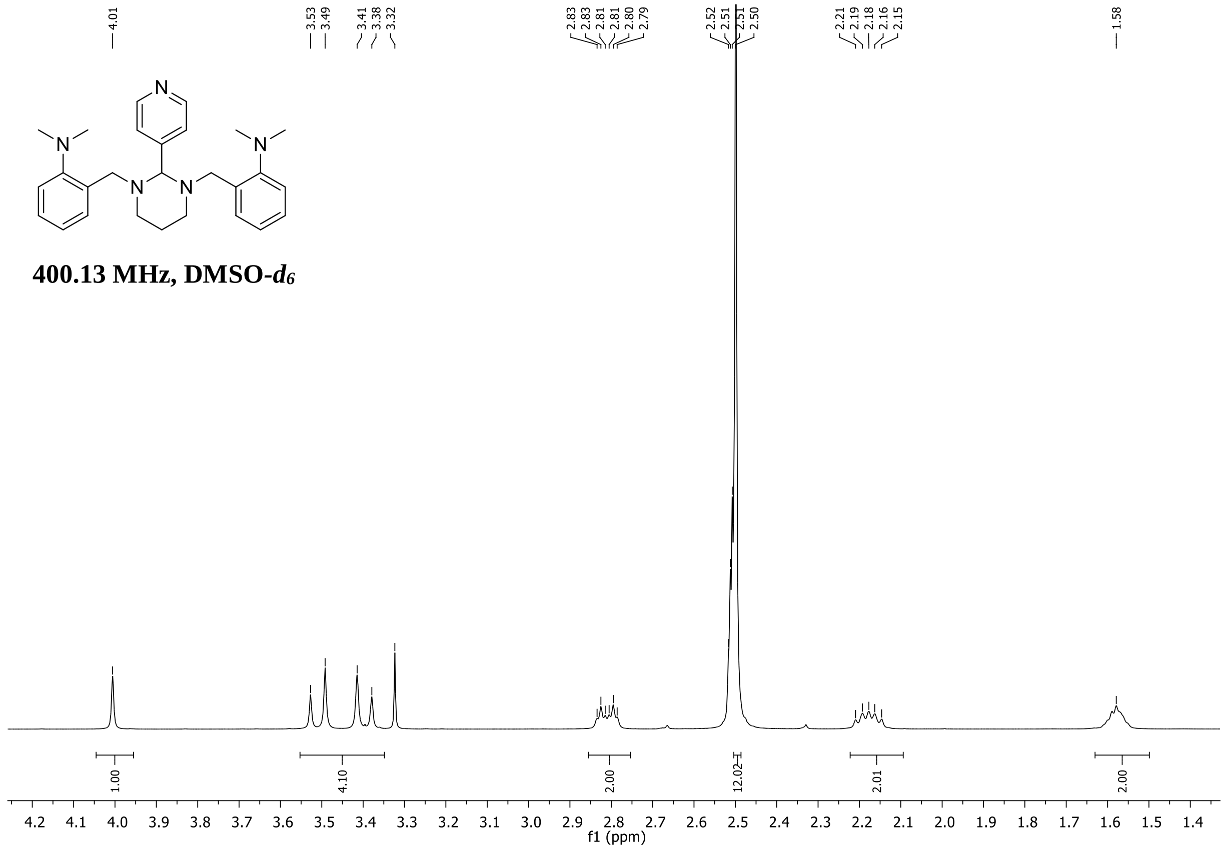

4.01
3.53
3.49
3.41
3.38
3.32
2.83
2.83
2.81
2.81
2.80
2.79
2.52
2.51
2.51
2.50
2.21
2.19
2.18
2.16
2.15
1.58
N
N
N
N
N
400.13 MHz, DMSO-d6
1.00
4.10
2.00
12.02
2.01
2.00
4.2
4.1
4.0
3.9
3.8
3.7
3.6
3.5
3.4
3.3
3.2
3.1
3.0
2.9
2.8
2.7
2.6
2.5
2.4
2.3
2.2
2.1
2.0
1.9
1.8
1.7
1.6
1.5
1.4
f1 (ppm)

^{13}C NMR of GANT61

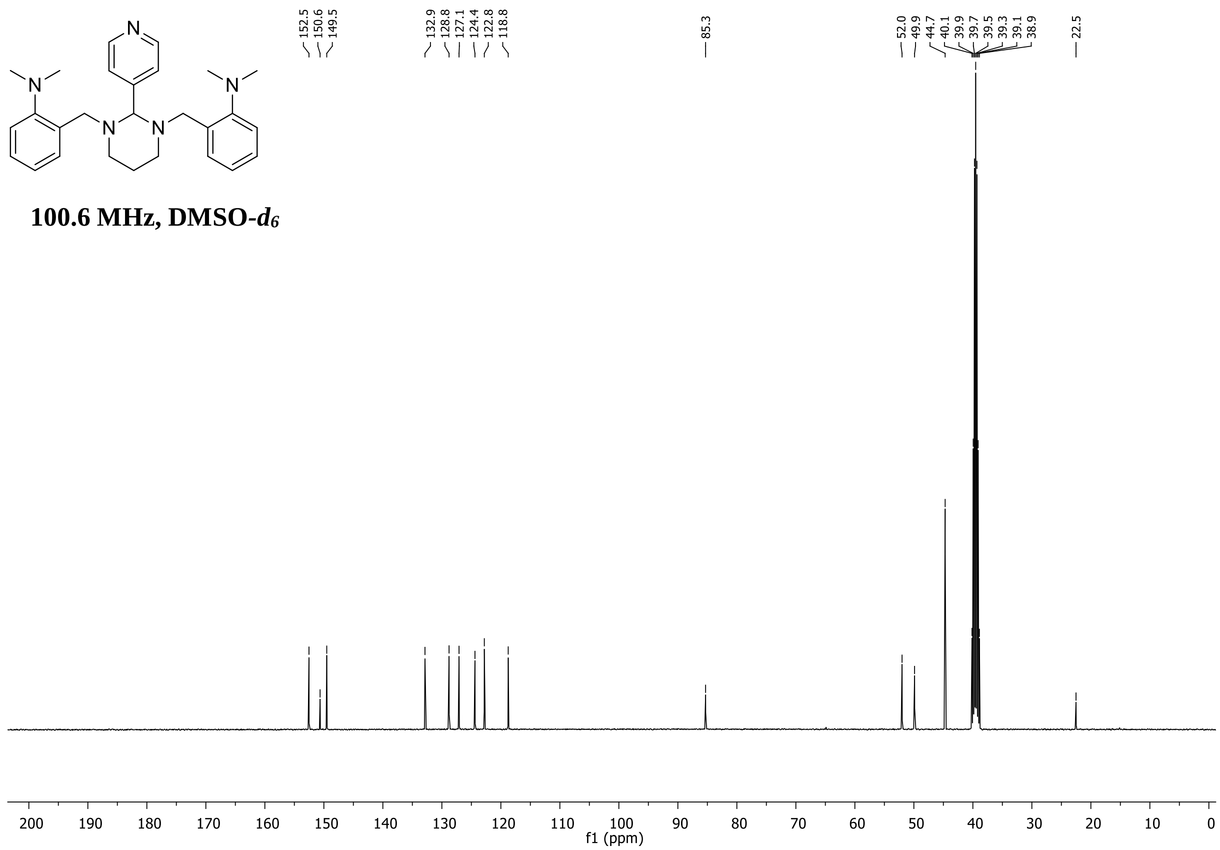

ESI MS of GANT61-D

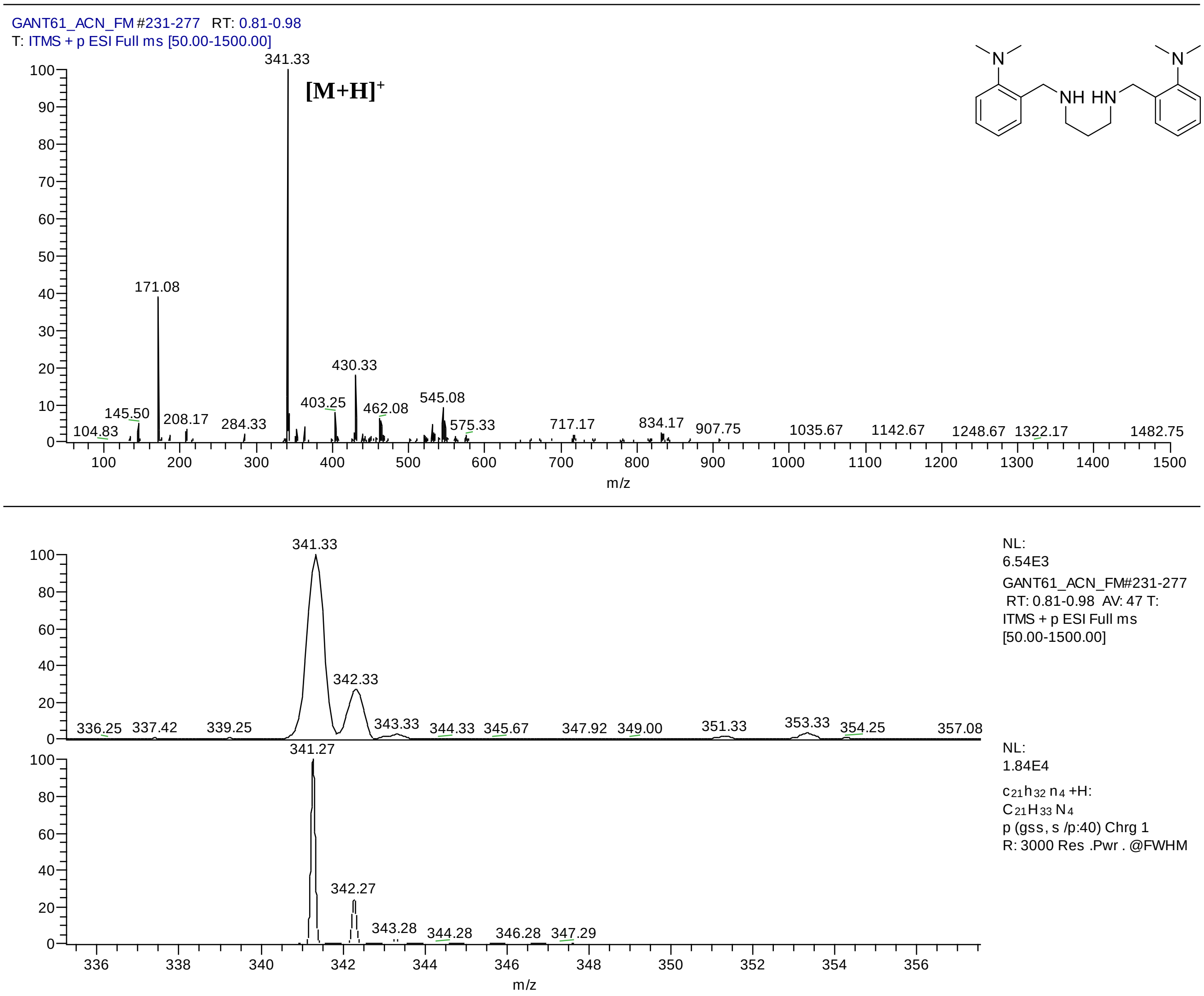

ESI MS of GANT61

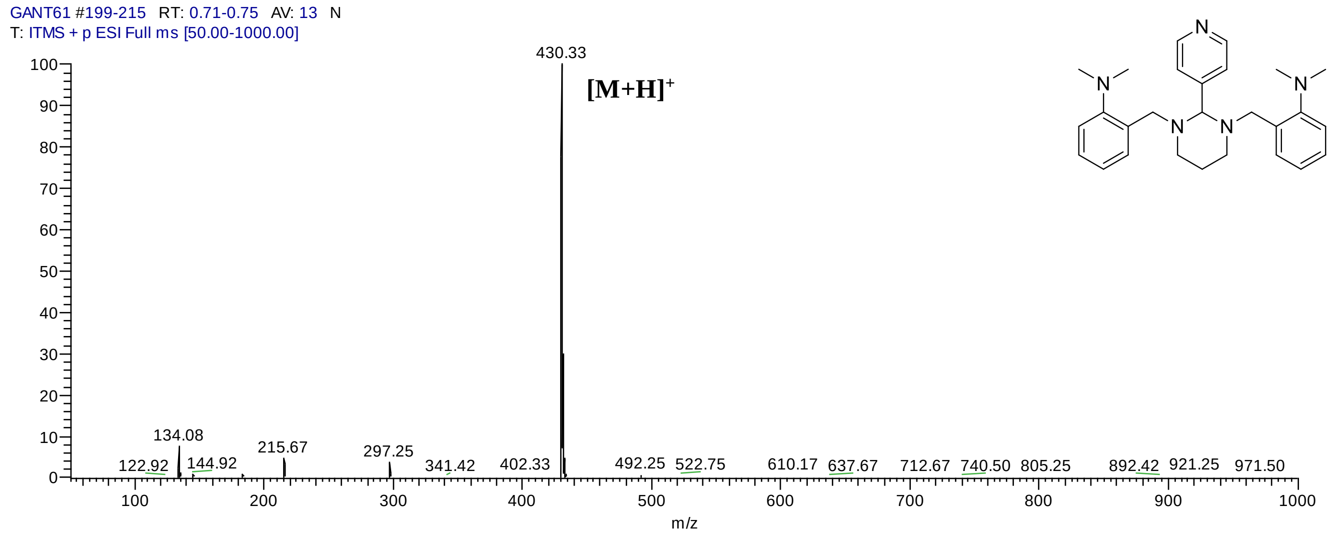

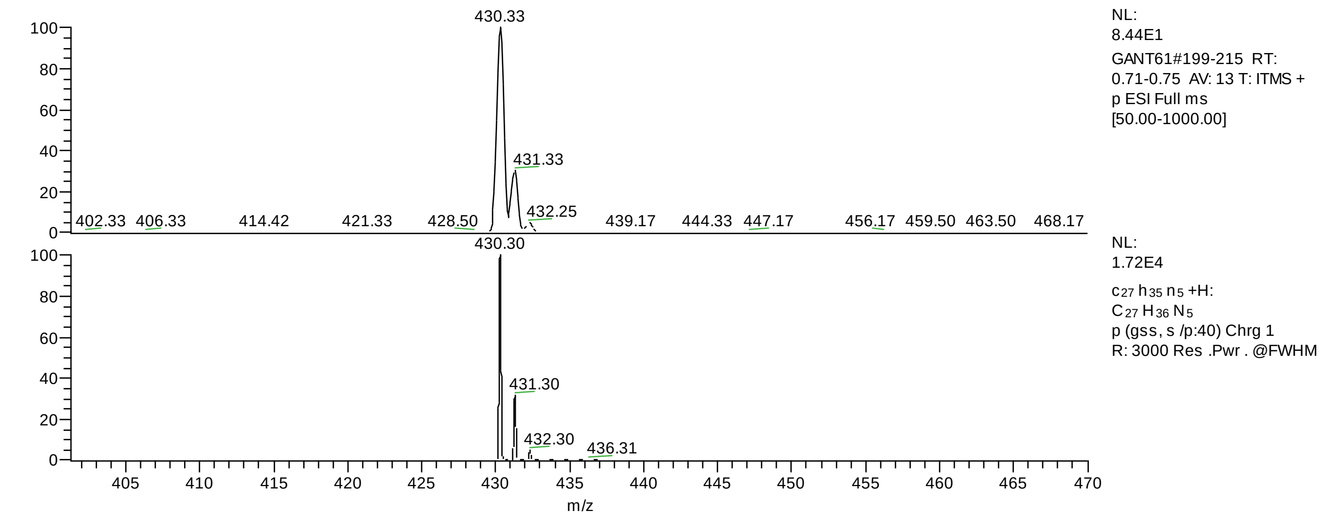

Supplement: IENZ_1419221_Supplementary_Material.pdf [file IENZ_A_1419221_SM2919.pdf]
